# Essential Oils as an Intervention Strategy to Reduce *Campylobacter* in Poultry Production: A Review

**DOI:** 10.3389/fmicb.2019.01058

**Published:** 2019-05-14

**Authors:** Andrew Micciche, Michael J. Rothrock, Yichao Yang, Steven C. Ricke

**Affiliations:** ^1^Center of Food Safety, Department of Food Science, University of Arkansas, Fayetteville, AR, United States; ^2^United States Department of Agriculture, Agricultural Research Service, Athens, GA, United States; ^3^Department of Poultry Science, University of Arkansas, Fayetteville, AR, United States

**Keywords:** *Campylobacter*, poultry, essential oils, oregano, thymol, carvacrol, cinnamaldehyde

## Abstract

*Campylobacter* is a major foodborne pathogen and can be acquired through consumption of poultry products. With 1.3 million United States cases a year, the high prevalence of *Campylobacter* within the poultry gastrointestinal tract is a public health concern and thus a target for the development of intervention strategies. Increasing demand for antibiotic-free products has led to the promotion of various alternative pathogen control measures both at the farm and processing level. One such measure includes utilizing essential oils in both pre- and post-harvest settings. Essential oils are derived from plant-based extracts, and there are currently over 300 commercially available compounds. They have been proposed to control *Campylobacter* in the gastrointestinal tract of broilers. When used in concentrations low enough to not influence sensory characteristics, essential oils have also been proposed to decrease bacterial contamination of the poultry product during processing. This review explores the use of essential oils, particularly thymol, carvacrol, and cinnamaldehyde, and their role in reducing *Campylobacter* concentrations both pre- and post-harvest. This review also details the suggested mechanisms of action of essential oils on *Campylobacter*.

## Introduction

*Campylobacter* is a leading cause of human gastroenteritis in the United States and worldwide (Corcionivoschi et al., 2012; Sibanda et al., 2018). In the United States alone, the Centers for Disease Controls and Prevention (CDC) estimates an annual 1.3 million cases occurring per year (Centers for Disease Control and Prevention [CDC], 2018). From 1999 to 2008, *Campylobacter* was estimated to cause an annual 8,463 hospitalizations and 76 deaths in the United States, along with an annual $1.7 billion financial cost to the United States (Batz et al., 2012). In 2015, of the 4,598 hospitalizations caused by foodborne disease 1,087 were the result of *Campylobacter* (Centers for Disease Control and Prevention [CDC], 2017a). This burden on public health was second only to *Salmonella* when considering bacterial infections (Batz et al., 2012; Scallan et al., 2015). When aggregating the loss of life and health due to illness, Scallan et al. (2015) determined that annually, *Campylobacter* caused 22,500 disability-adjusted life years (DALY). *Campylobacter* was ranked 3^rd^ as the leading impact of public health due to foodborne illness behind non-typhoidal *Salmonella* (32,900 DALY) and Toxoplasma (32,700 DALY). A systematic analysis from 1990 to 2013 identified *Campylobacter* as the fourth leading cause of diarrheal disease behind rotavirus, typhoid fever, and cryptosporidiosis (Murray et al., 2015). According to the World Health Organization (WHO), diarrheal diseases, viewed as a whole, cause an estimated 550 million illness each year resulting in the annual death of approximately half a million infants under the age of two (World Health Organization [WHO], 2013).

In a population-based study on patients with diarrheal illness induced by *Campylobacter*, over 95% of the 1,316 cases were caused by *Campylobacter jejuni* (Friedman et al., 2004). This species, along with pathogen *Campylobacter coli*, have been found in the gastrointestinal tract (GIT) of poultry (Wang et al., 2002; European Food Safety Authority [EFSA], 2010; Centers for Disease Control and Prevention [CDC], 2017b). Broilers and layer flocks have consistently been shown to contain *Campylobacter* prevalences greater than 70% (Corry and Atabay, 2001; Stern et al., 2001; Ansari-Lari et al., 2011; Sahin et al., 2015). Schets et al. (2017) found that 97% of layer and 93% of broiler flocks tested positive for *Campylobacter*. Across eight flocks in the Netherlands, 55 cecal samples were taken and found that *C. jejuni* was the *Campylobacter* isolate for 100% of cecal broiler samples where in layer hens 52% were *C. coli*, 40% were *C. jejuni* (40%) and 7% were *C. lari*. Identical sequence types were found in the soil, sediment, and surface water which indicates potential contamination of the bird through environmental means. Through contamination of the poultry carcass, *Campylobacter* can cause foodborne illness and has been the etiological agent in outbreaks caused by poultry products (Nauta et al., 2009; Geissler and Powers, 2011). Therefore there is a continued urgency to implement pre- and post-harvest technologies to prevent *Campylobacter* contamination at all stages of poultry production.

With the rise of multidrug-resistant bacteria, consumer preference for antibiotic-free chicken, and government regulations such as the European Union’s 2006 ban of antibiotics, alternative antimicrobials have become necessary (Cervantes, 2015; Johnson, 2015; Shin et al., 2015). One alternative to conventionally implemented pre- and post-harvest antimicrobial strategies is the use of essential oils (EOs) (Tiihonen et al., 2010; Amerah et al., 2012; Calo et al., 2015; O’Bryan et al., 2015; Thibodeau et al., 2015; Micciche et al., 2018b). The EOs industry had a United States market share of $6.6 billion in 2016, a 286% increase from the 2004 market share of $2.3 billion (Zviely, 2004; Grand View Research, 2018). They are often used as flavoring agents in food products and perfumes, embalmment, anti-inflammatory and anesthesia remedies (Burt, 2004; Bakkali et al., 2008). Some EOs, such as eugenol, have been reported to have a preventative effect against cancer (Burt, 2004; Tsuneki et al., 2005; Bakkali et al., 2008). Plant-based EOs such as eugenol, thymol, carvacrol, and cinnamaldehyde have been examined and screened for their antimicrobial properties against a number of pathogens including *Campylobacter* (Friedman et al., 2002; Chouliara et al., 2007; Thibodeau et al., 2015; Kelly et al., 2017; Upadhyay et al., 2017). They are also commercially attractive as alternative antimicrobials, because they are considered acceptable for organic and non-conventional applications (Micciche et al., 2018b; National Organic Program, 2018). They may be useful not only in preventing human health-related diseases but in improving the performance of the bird (Alcicek et al., 2004; Hernandez et al., 2004; Diaz-Sanchez et al., 2015; National Organic Program, 2018). As such, the objective of this review was to discuss EOs as an intervention approach for limiting *Campylobacter* contamination in the poultry industry pre- and post-harvest. By investigating the metabolic activity of *Campylobacter*, a greater understanding of the mechanistic effects of EOs may be elucidated for more optimal application approaches in the future.

## *Campylobacter* Characteristics and Metabolism

*Campylobacter* belongs to family Campylobacteraceae, which also includes *Arcobacter* and *Helicobacter* (Fitzgerald and Nachamkin, 2011). They are non-sporulating Gram-negative microorganisms, with an s-shape and cell dimensions between 0.5 and 5 μm and ranging in width from 0.2 to 0.9 μm (Vandamme et al., 2006). A single polar flagellum is found in 20 of the 22 species with the exception being the non-motile *C. gracilis* and *C. showae* that possess multiple flagella (Debruyne et al., 2008; Facciolà et al., 2017). *Campylobacter* spp. are microaerophilic and grow optimally in a gas composition of 5% O_2_, 10% CO_2_, and 85% N_2_, pH of 6.5 to 7.57 and from 37 to 42°C (Garénaux et al., 2008; Davis and DiRita, 2017; Facciolà et al., 2017). *Campylobacter* will not grow at temperatures below 30°C, due to an absence of a cold shock protein gene, or with a water activity below 0.987, which is necessary to maintain turgor pressure (Hazeleger et al., 1998; De Cesare et al., 2003; Wallace, 2003; Levin, 2007; Facciolà et al., 2017). Cold shock proteins range from 65 to 75 amino acids serve as nucleic acid chaperones that prevent mRNA secondary structures from forming which enable efficient transcription and translation (Phadtare and Severinov, 2010; Keto-Timonen et al., 2016). Interestingly, despite the lack of a cold shock protein, biofilms of *C. jejuni* have been found to form and persist in 13°C conditions and formed biofilms with the largest surface area compared to 20, 37, and 42°C (Sanders et al., 2008). Attachment of planktonic cells to the biofilms was not significantly impacted by temperature (Sanders et al., 2008).

Compared to other foodborne pathogens occupying the poultry GIT, *Campylobacter* possesses a metabolism that is somewhat different and thus may be a challenge for some intervention strategies. *Campylobacter* spp. do not utilize the traditional glycolysis pathway, instead favoring amino acids such as aspartate, glutamate, serine, and proline for their cellular respiration pathways (Stahl et al., 2012). The traditional glycolytic pathway is incomplete as genes encoding glucokinase and 6-phosphofructokinase are absent (Parkhill et al., 2000). Furthermore, despite a complete tricarboxylic acid cycle (TCA), gluconeogenesis from glucose-6-phosphate to glucose is not observed, and the genes necessary for encoding the proteins associated with the pathway are absent (Parkhill et al., 2000; Velayudhan and Kelly, 2002). Some of the gluconeogenesis and glycolysis genes and respective proteins are present which has led to the hypothesis that *C. jejuni* can catabolize intermediary molecules (Stahl et al., 2012). For instance, Hofreuter et al. (2006) suggested that the presence of a glycerol-3-phosphate transporter (GlpT) in *C. jejuni* 81176 could be indicative of the breakdown of glycerol-3-phosphate to generate pyruvate and potential further breakdown through the TCA cycle. Additionally, the non-oxidative portion of the pentose pathway shunt (transaldolase, transketolase, ribulose-3-phosphate epimerase, and ribose-5-phosphate isomerase) is suggested to have the ability to metabolize pentose sugars (Line et al., 2010).

Two studies independently identified novel l-fucose pathways with a l-fucose permease that is homologous to the one characterized in *E. coli* (Dang et al., 2010; Muraoka and Zhang, 2010; Stahl et al., 2011). Fucose is a component of eukaryotic glycoproteins and is found in mucin (Allen and Griffiths, 2001; Robbe et al., 2004). *Campylobacter jejuni* utilizes fucose as a chemoattractant for mucin attachment (Hugdahl et al., 1988; Tu et al., 2008; Gangaiah et al., 2010; Kassem et al., 2013). While fucose uptake was phenotypically observed in *C. jejuni* strain NCTC 11168 (isolated from human feces), it was not detected in strains 81116 and 81176 (Muraoka and Zhang, 2010). This is due to the presence of a genomic island cj0480c–cj0490, which has been linked to an increase in virulence (Parker et al., 2006). Stahl et al. (2011) noted that this metabolic pathway improved colonization of the piglet model for human disease indicating its importance in human-associated pathogenesis. Moreover, when the l-fucose pathway genes were mutated, *C. jejuni* was still able to colonize the ceca of chickens but was outcompeted in a co-colonization experiment by the wild-type strain (Muraoka and Zhang, 2010). Despite the presence of this metabolic pathway, *Campylobacter* is largely asaccharolytic and relies primarily on amino acids and organic acids for its energy and carbon needs (Lin et al., 2009; Stahl et al., 2012; Kassem et al., 2013; Kelly et al., 2017).

Amino acids utilized by *Campylobacter* as carbon and energy sources include asparagine, glutamine, serine, aspartate, and proline (Wright et al., 2009). *Campylobacter jejuni* will preferentially utilize serine, aspartate, asparagine, and glutamate (Guccione et al., 2008; Wright et al., 2009; Stahl et al., 2012). Parsons (1984) determined the most common amino acids within the ceca of leghorn hens through ion-exchange chromatography. Serine ranked 4^th^ (92.2 mmol/mol ceca content), aspartate ranked 2^nd^ (109.9 mmol/mol ceca content), and glutamate ranked 1^st^ (137.5 mmol/mol ceca content) as the most concentrated amino acids within the ceca. Asparagine, glutamine, glycine, cysteine, and tryptophan concentrations were not reported. While these concentrations will vary depending on diet, GIT microbial population, and bird type, the data provides insight into why amino acid metabolism of *C. jejuni* could play an essential role in its ecological niche within the poultry GIT (Ravindran and Bryden, 1999; Stahl et al., 2012). Whereas other species must rely on carbohydrate fermentation, the plentiful concentration of amino acids in the poultry ceca, due to protein-rich diets, allows *Campylobacter* to thrive (Józefiak et al., 2004; Vegge et al., 2009; Hermans et al., 2012).

Not all poultry dietary components contribute directly to the nutrition of the bird but may still interact with the GIT microbial population including resident foodborne pathogens. For example, there are primary plant compounds and secondary plant metabolites present in the poultry GIT that may not necessarily serve directly as nutrients but can still nutritionally influence bird performance (Smithard, 2002). While some fiber components, particularly lignin, are generally considered indigestible in the avian GIT, they can still impact GIT microbial composition, alter fermentation patterns, and influence metabolite absorption (Ricke et al., 1982, 2013; Jung and Fahey, 1983; van der Aar et al., 1983; Dunkley et al., 2007; Baurhoo et al., 2008; Sima et al., 2018). These high fiber sources have also been shown to limit the establishment of foodborne pathogens such as *Salmonella* Enteritidis in laying hens when serving as the primary dietary source in laying hens (Ricke, 2003; Woodward et al., 2005; Ricke et al., 2013). Plants also contain phenolic monomers (Jung and Fahey, 1983). Phenolic monomers can not only cross-link carbohydrates with lignin in the plant, decreasing fiber degradation in the GIT, but in their free form can be antimicrobial to aerobic and anaerobic bacteria (Zemek et al., 1979; Jung and Fahey, 1983).

Aromatic compounds can also be modified anaerobically (Tschech and Schink, 1985; Netzer et al., 2016). Often phenolic compounds such as chlorophenol and chlorobenzoate can be utilized as electron sinks, resulting in their reduction and modification within the lower GIT (Häggblom et al., 1993; Frazer, 1994). While *Campylobacter* has been shown to utilize some aromatic compounds such as resorcinol and β or γ-resorcylate, this was only shown in the presence of *Clostridium* spp. (Tschech and Schink, 1985; Evans and Fuchs, 1988). However, other aromatic compounds can be utilized as energy sources by some bacteria (Overhage et al., 2006). MetaCyc identifies five complete aromatic degradation pathways present in *E. coli* including cinnamate, phenylethylamine, nitroaromatic, and phenols (version 22.5; BioCyc; Menlo Park, CA, United States) (Caspi et al., 2015). Chlorobenzene, paraoxon, parathion, and shikimate degradation pathways have been identified in *L. monocytogenes* and degradation of *p*-cymene, along with other aromatic compounds, have been characterized in *Pseudomonas* spp. In *Campylobacter*, only the catechol degradation pathway has been detected. Numerous biodegradation enzymes have been elucidated in *E. coli;* for instance, eugenol has been shown to be degraded to ferulic acid by the *vaoA* gene-encoded enzymes (Díaz et al., 2001; Overhage et al., 2003, 2006). However, only resorcinol, protocatechuate, and phloroglucionol degradation enzymes are known to occur in *Campylobacter* (Evans and Fuchs, 1988; Villemur, 1995). Given the limited metabolic capacity of *Campylobacter* for modification, it would be intuitive that aromatic compounds that exhibit antimicrobial properties could effectively reduce *Campylobacter* populations in poultry production. This is important since aromatic compounds are one of the constituents present in phytobiotics.

## Phytobiotics

Phytobiotics are plant-based compounds or extracts that have been suggested for use in commercial, and possibly organic, poultry production (Windisch and Kroismayr, 2007; Bakkali et al., 2008; Diaz-Sanchez et al., 2015; Micciche et al., 2018b). Most phytobiotics are Generally Recognized As Safe (GRAS) by the U.S Food and Drug Administration [FDA], 2018 and are less toxic and typically more residual-free compared to synthetic antibiotics (Diaz-Sanchez et al., 2015; U.S Food and Drug Administration [FDA], 2018). Botanicals, a subset of phytobiotics, are leaves, roots, bark, or other parts of a plant, and the terminology is often used interchangeably with phytobiotics (Windisch and Kroismayr, 2006; Mohammadhosseini et al., 2017). Other types of phytobiotics include herbs, which are derived from flowering non-persistent plants, oleoresins which are non-aqueous extracts such as balsam, and EOs (Prior and Cao, 2000). In this review, the focus will primarily be on the activity of EOs, but other phytobiotic compounds such as herbs will also be mentioned.

Essential oils, also known as volatile or ethereal oils, are oily plant-based liquids that possess aromatic properties (Burt, 2004; Hardin et al., 2010). The term “essential oils” was first coined by Paracelsus von Hohenheim in the 16^th^ century (Guenther, 1948). The term ‘essential’ relates to the effective element in a medical preparation of the drug and is therefore loosely defined (Oyen and Dung, 1999). Currently, there are over 3,000 known EOs with approximately 300 being commercially relevant (Bakkali et al., 2008; Diaz-Sanchez et al., 2015). They include oils such as turpentine, eugenol, and cinnamaldehyde, and can be derived from other botanical compounds and herbs such as thyme, oregano, rosemary, and lemon (Burt, 2004; Fisher and Phillips, 2008; Diaz-Sanchez et al., 2015). Essential oils are extracted from their corresponding plants by steam distillation, hydrodistillation, or solvent extraction which all can create a concentrate of aromatic and volatile compounds including terpenoids and phenylpropanoids (Nakatsu et al., 2000; Hardin et al., 2010; Raut and Karuppayil, 2014). The concentrations of the ‘essential’ compounds in EOs vary wildly and are not typically defined (Lee et al., 2004a; Benavides et al., 2012).

## Non-Antimicrobial Effects of EOs in Poultry

Essential oils have a wide range of applications including turpentine for paint mixing or linalool and linalyl acetate that are used as alternative sleep aides (Buchbauer et al., 1991; Burt, 2004). Many are incorporated as ingredients for their palatable tastes and smells in foods and aromatic sprays (Franz et al., 2010). Oregano, thyme, and cinnamon are well-known flavor enhancers (Khan and Abourashed, 2011; Wang et al., 2013). Bergamon is used for its aromatic properties of Earl Gray Tea, while synthetic based citrus oils are an ingredient in soft drinks (Fabricant, 2008; Gonzalez-Molina et al., 2009; Callaway et al., 2011). While 32.9% of EOs are applied in the food and beverage industry, the second largest application of EOs in 2015 was for spa or relaxation purposes (30.84%) (Grand View Research, 2018).

In poultry, EOs have been utilized in preharvest management settings for non-pathogen related benefits (Diaz-Sanchez et al., 2015). Digestibility of poultry feed has been shown to be improved by the addition of EOs (Williams and Losa, 2001). CRINA^®^ (Akzo Nobel, Crina S.A, Switzerland) is a commercial blend of EOs containing thymol, eugenol, and piperine. Broilers fed 50 mg/kg of CRINA^®^, exhibited improved activity of total amylase, trypsin, and maltase of 40kU/pancreas, 63U/pancreas, and 12.6 μM/g mucosa respectively, compared to the control group activity of 29kU/pancreas, 42U/pancreas, and 10.6 μM/g mucosa, respectively (Jang et al., 2004, 2007). This effect was not observed; however, when 0.1% of lactate was also introduced into the diet (Jang et al., 2004). The use of 200 ppm of a blend of oregano, cinnamon, and pepper improved fecal digestibility of dry matter (Hernandez et al., 2004). Ileal absorption levels of amino acids such as threonine, serine, asparagine, phenylalanine, histidine, and lysine were positively improved by 7.8 and 8.8% by the addition of 150 and 300 ppm of a plant extract containing capsaicin, carvacrol, and cinnamaldehyde (Jamroz et al., 2003).

Amino acid absorption studies have also been performed using rat intestines. A catheter was attached to both ends of a sector of the jejunum of anesthetized rats (Kreydiyyeh et al., 2000). In the treatment groups, the jejuna were preincubated with 1000 ppm cinnamaldehyde or 850 ppm of eugenol or a saline control. Alanine was then fed through the jejuna, and it was observed that after 40 min the jejuna preincubated with cinnamaldehyde and eugenol absorbed 22 to 25 nmol of alanine compared to 60 nmol in the control. This suggests reduced nutrient absorption (Kreydiyyeh et al., 2000; Lee et al., 2004a). However, conclusions drawn from this study may be difficult to apply to production animals due to the high concentrations necessary in the feed to result in similar concentrations in the GIT as utilized in Kreydiyyeh et al. (2000). Lee et al. (2003) observed that 100 ppm of thymol or cinnamaldehyde exhibited no significant effect on pancreatic digestive enzyme activity at day 21 or day 40 within female broilers (Lee et al., 2004a). This study shows that thymol alone is unable to impact pancreatic digestive enzymes within poultry and a combination of multiple EOs, such as in Jang et al. (2004, 2007), may be necessary to impact digestibility responses in broilers. However, it may be specific combinations of EOs that impact digestibility and nutrient absorption.

Improvements in nutrient absorption and digestibility can result in improved growth rates within broilers (Gous, 2010). Feed conversion ratio (FCR), the ratio between feed intake and average weight gain, is one of the more commonly used metrics to determine if a feed additive is beneficial to commercial livestock production (Leenstra, 1986). Several studies have reported no improvement in growth rate or FCR when diets have been supplemented with EOs (Lee et al., 2003; Jang et al., 2004, 2007). However, FCR and body weight gain (BWG) improvements have been observed in other studies that utilized EOs (Denli et al., 2004; Cabuk et al., 2006; Basmacioğlu Malayoğlu et al., 2010). Cabuk et al. (2006) demonstrated that 24 or 48 mg/kg of Heryumix^TM^ significantly improved FCR on days 21 to 1.53 and 1.56 compared to the control at 1.62. On day 42, compared to the control at 1.87, the FCR was also improved to 1.80 and 1.77 with 24 or 48 mg/kg of Heryumix^TM^ (Cabuk et al., 2006). Heryumix^TM^ is composed of oregano, laurel leaf, sage, myrtle, fennel, and citrus peel extracts (Herba Gida Maddeleri; Seferihisar, Turkey). Denli et al. (2004) observed similar improvements in quail when their diet was supplemented with 60 mg/kg of thyme or black seed oil.

This discrepancy may be due to certain variables including bird type, feed composition, and EO type. The intestinal mucosa and GIT microbiome composition of birds vary depending on the species and age of the bird (Zoetendal et al., 2004; Stanley et al., 2014). While the phyla *Bacteroidetes* and *Firmicutes* are dominant within quail and broiler chicken cecal microbiomes, the represented genera are different (Oakley et al., 2014; Liu et al., 2015). Over 117 different genera were identified by Wei et al. (2013) in broiler chicken ceca. The quail microbiome, however, is not as taxonomically rich at the genus level with only 32 genera being detected by Wilkinson et al. (2016). Furthermore, while the top five most dominant genera based on taxa analyses were *Lactobacillus, Rumiunoccocus, Clostridium, Bacteroides, Faecalibacterium* in the chicken cecal microbiome, the quail ceca microbiome was dominated by *Bacteroides, Ruminococcus, Faecalibacterium, Enterococcus*, and *Clostridium* (Wei et al., 2013; Wilkinson et al., 2016). These microbiome differences along with unknown host-species interactions should be considered when evaluating and comparing the effectiveness of any particular feed amendment across species (Koutsos and Arias, 2006).

Additionally, the feed may play a major role in how EOs improve FCR. As elucidated in Jamroz et al. (2003), EOs can impact the absorption of amino acids and potentially other nutrients in the ileum. As a consequence, the FCR should be improved in birds on typical commercial diets amended with EOs. Furthermore, improved absorption of amino acids could conceivably also allow for the use of non-conventional diets that contain less protein. In-depth studies employing not only commercial bird performance measurements but intestinal pathway and tissue profiling to screen and compare feed composition and EO blend combinations would be necessary to address this hypothesis.

Finally, while terpenoids are the main constituents of EOs, their chemistry can vary widely (Jager, 2010). For instance, carvacrol is a monoterpene alcohol and, in rats, its aliphatic group readily undergo aromatic hydroxylation while its alcohol group undergoes carboxylation (Jahrmann, 2007; Jager, 2010). Thymol on the other hand forms derivatives of benzyl alcohol and 2-phenylpropanol when reduced (Austgulen et al., 1987). When carvacrol was fed to rats, their excreted urine contained more of 2-(3-Hydroxy-4-methylphenyl)propan-2-ol then carvacrol but when thyme was fed to rats it was in the highest concentration out of its five derivates (Austgulen et al., 1987). This suggests, in rats, that carvacrol undergoes chemical interactions and is metabolized more than thymol. This functional difference between EO metabolism, along with the importance of bird type and feed composition, may in part explain the variation in EOs benefit to broiler nutrition.

Although there is variation in EOs benefit to poultry growth, the antioxidant activity of many EOs is well-known (Baratta et al., 1998; Ruberto et al., 2000; Martucci et al., 2015). Rosemary oil, thymol, carvacrol, oregano, ginger, and coriander all possess antioxidant activity (Wei and Shibamoto, 2007). An oxidation deterioration test involving the application of EOs to lard indicated that 0.20% oregano possessed the most antioxidant capacity followed by thyme, dittany, marjoram, spearmint, then lavender (Economou et al., 1991). Economou et al. (1991) also found that combinations of thyme and marjoram and thyme and spearmint EOs also had potential synergistic properties in protecting lard from oxidation. These properties benefit bird health and can be marketable if EOs are used on the finished product (Lee et al., 2004b; Diaz-Sanchez et al., 2015).

Additionally, the sensory characteristics of EOs mean that they can enhance the sensory characteristics of the final products if used in the appropriate concentrations. The addition of 300 mg/kg of oregano, garlic, or an equal combination in the diets was shown to significantly improve the flavor of frozen chicken breasts up to 60 days (Kirkpinar et al., 2014). Birds fed the EOs were processed, and the breasts were stored at -25°C for sensory analysis. Overall flavor evaluated on days 1, 15, and 30 indicated that all EOs treatments scored significantly higher than the control. Overall acceptability scores of breast meat indicated that only garlic resulted in a more palatable final product on days 1, 15, and 30. Overall acceptability scores were based on flavor, appearance, and tenderness. Days 45 and 60 scores were not analyzed statistically due to spoilage. On organic seabass filets, the addition of 0.2% thyme oil improved the sensory characteristics (Kostaki et al., 2009). A panel of seven judges evaluated the filets on nine days within a 21-day storage trial where the filets were held at -30°C. While the control group reached the acceptability limit on taste in 6 days the addition of 0.2% thyme improved the sensory characteristics scores extending shelf life by 2 days. When the filets were placed in modified atmospheric packaging (MAP) (60% CO_2_; 30% N_2_; 10% O_2_), sensory characteristics were improved to allow for a shelf life of 14 days. When thyme oil was added to the MAP, shelf life was extended by 3 days. Despite these potential benefits, their antimicrobial activities might be some of their more important attributes to commercial poultry production.

## Antimicrobial Mechanisms of Essential Oils

### Indirect Antimicrobial Mechanisms

There are indirect characteristics associated with the presence of EOs that may play a role in reducing *Campylobacter* and other pathogen loads on the final poultry meat product. While no definitive mechanism has been elucidated, there have been several potential antimicrobial outcomes that may indirectly impact *Campylobacter*. For instance, the improved ileal absorption of amino acids within broilers, as demonstrated by Jamroz et al. (2003), may limit a required nutrient source for *Campylobacter* in the ceca (Velayudhan et al., 2004). Improvement of the immune response may also impact pathogen concentrations (Diaz-Sanchez et al., 2015). Layer hens exhibited improved antibody titer levels to Newcastle disease and infectious bursal disease when their diets were supplemented with Heryumix^TM^ (Özek et al., 2011). In another study with Heryumix^TM^, the humoral immune response of layer hens in heat stress was not stimulated (Bozkurt et al., 2012). Basmacioğlu Malayoğlu et al. (2010), noted that broilers fed 0, 250, or 500 mg/kg of oregano exhibited IgG concentrations of 27.42, 30.50, and 39.41 mg/dL, respectively. The IgM concentrations were 7.91, 9.58, and 11.71 mg/dL, respectively (Basmacioğlu Malayoğlu et al., 2010). However, while these concentrations of antibodies were higher, they were not statistically significant (*P* > 0.05) (Basmacioğlu Malayoğlu et al., 2010). As such, further research should be conducted to elucidate the mechanism(s) of EOs on the poultry immune system. Understanding how EOs improve the immune system of poultry may be important because *Campylobacter* colonization may elicit an immune response, and therefore there is potential to reduce *Campylobacter* concentrations through immune system modulation (Connerton et al., 2018). Essential oils may also interact with *Campylobacter* populations directly.

### Direct Antimicrobial Mechanisms

Much of the mechanisms associated with antimicrobial activities of EOs have been elucidated from microorganisms other than *Campylobacter*, and thus assumptions regarding *Campylobacter* must be inferred to some extent. General antimicrobial mechanisms associated with EOs have been extensively described previously by O’Bryan et al. (2015) and will be discussed briefly in the current review with specific emphasis on *Campylobacter* where applicable. Essential oils have been shown to alter proteomes and cell morphology of pathogenic bacteria (Nazzaro et al., 2013; O’Bryan et al., 2015). Significant morphological differences in cell shape have been observed when EOs such as mint, thymol, and cinnamaldehyde have been applied to bacteria (Kwon et al., 2003; Kalchayanand et al., 2004; Hajlaoui et al., 2009). For instance, the use of cinnamaldehyde on *Bacillus cereus* inhibited cell division resulting in elongated filamentous cells that were clumped together with incomplete septa (Kwon et al., 2003). After 1 h, almost all cells were in filamentous chains with no clear septas. *Salmonella enterica* serovar Thompson grown in the presence of a sub-lethal concentration of thymol (0.01%) demonstrated an altered proteomic profile compared to the control, which included downregulation of binding and chemotaxis proteins, but resulted in upregulation of other outer membrane proteins (Di Pasqua et al., 2010). In-depth analysis using 2-D PAGE, followed by MALDI-TOF, revealed that GroEL and DnaK were upregulated in the presence of thymol. GroEL, along with GroES, as well as DnaK, along with DnaJ, prevents misfolding and proper indiscriminate assembly of polypeptides under stress conditions within the cytoplasm (Fenton and Horwich, 1997; Motojima, 2015). Changes in regulation were detected by spot detection, and the relative size of the GroEL spot was 0.109 units in the control compared to a spot size of 1.044 units. The DnaK spot was not detected in the control but exhibited a size of 0.267 in the treatment with thymol. Other downregulated proteins include CheW, which is involved in transferring sensory signals from chemoreceptors to flagellar motor proteins, and thioredoxin docking proteins (Trx1). The spot size of CheW was 0.153 units and was not detected in the thymol treated cells where the Trx1 spot was 0.223 units in the control and not detected in the thymol treatment. Trx1 is an active oxidation–reduction protein that has been found to be involved in cell division in *E. coli*, which suggests thymol may play a role in the inhibition of bacterial population growth (Kumar et al., 2004). Trx1 refolds citrate synthase, an essential enzyme in the TCA cycle, and by downregulating Trx1 with thymol, citrate synthase was not present. In addition, enzymes of the reverse TCA cycle were upregulated in the thymol treatment such as an increase of citrate lyase from 0.08 units to 0.654. Acetate kinase was also reduced from 0.567 units to 0.19 units within *Salmonella*. These results may not be replicated in *Campylobacter* due to its incomplete glycolytic pathways and studies investigating the use of EOs to alter the proteome of *Campylobacter* should be performed.

Another proposed mechanism for the effect of EOs on bacteria such as *Campylobacter* is their potential to disrupt the outer membrane and initiate cell lysis (O’Bryan et al., 2015). Attributed largely to EOs hydrophobicity, the outer membrane lipids may be disrupted, sheared, or penetrated, allowing for an increase in permeability (Fisher and Phillips, 2009; Brenes and Roura, 2010; Guinoiseau et al., 2010). Carvacrol and thymol, in 200 mg/L concentrations, have been demonstrated to inhibit *E. coli* through fluorescent flow cytometry (Xu et al., 2008). The mechanism proposed in this study, supported by Helander et al. (1998), was that EOs disrupt the lipopolysaccharides membrane structure and alter the proton gradient (Xu et al., 2008). This effect may not occur in *Campylobacter* spp. due to their reliance on fermentation pathways and would have to be investigated (Line et al., 2010). Alterations of the lipopolysaccharide membrane can still lead to disruption of the cytoplasmic membrane and cell lysis (Xu et al., 2008). Electron microscopy has demonstrated the *E. coli* treated with oregano oil resulted in cell membrane collapse and leakage of contents (Sikkema et al., 1995; De Sousa et al., 2012). Cumin derived *p*-cymene has been demonstrated to swell bacterial cell membranes and has been suggested to be used synergistically with carvacrol to lyse bacterial membranes (Ultee et al., 2002). Other phenolic compounds have been observed to demonstrate this effect on the bacterial membrane (Cosentino et al., 1999; Juliano et al., 2000; Lambert et al., 2001; Brenes and Roura, 2010).

Essential oils have also been shown to impact Gram-positive organisms (Si et al., 2006). Cinnamaldehyde and eugenol, only when used in combination, were shown to inhibit *Staphylococcus, Micrococcus*, and *Bacillus* (Moleyar and Narasimham, 1992). Moreover, when considering the 300 commercially viable EOs, it is quite likely not all EOs may operate under the same mechanism (Diaz-Sanchez et al., 2015). They may even operate in concert with a series of mechanisms that represent contradictory activities against bacterial cellular processes. While some EOs blends promote the growth of beneficial bacteria others have inhibited beneficial bacteria such as *Lactobacillus*, and even some *Bacillus* species (Kivanç et al., 1991; Manzanilla et al., 2001; Delaquis et al., 2002; Jamroz et al., 2003; Donsi et al., 2011). Furthermore, the antimicrobial activity may not be attributable to one specific mechanism (Skandamis et al., 2001; Carson et al., 2002). When considering all of the proposed mechanisms it seems more than likely that multiple mechanisms are responsible for the effect of EOs against pathogens including *Campylobacter* (Diaz-Sanchez et al., 2015). As a consequence, the impact of EO blends on *Campylobacter* populations may vary considerably in pre and post-harvest applications.

## Antimicrobial Effects of EOs on *Campylobacter* in Poultry Pre-Harvest Environments

### Transmission and Colonization of *Campylobacter* in the Poultry GIT

The use of EOs in pre-harvest environments has focused on preventing pathogen colonization or reducing their concentration in the GIT (Brenes and Roura, 2010). *Campylobacter* resides in the intestinal mucosa of the avian GIT and can be rapidly transmitted throughout a flock via the drinking water and fecal-oral route (Montrose et al., 1985; Beery et al., 1988; Keener et al., 2004). *Campylobacter* virulence factors that impact colonization include, *motA, fliA, jlpA*, and *racR* and were discussed in Upadhyay et al. (2017) and reviewed in Bolton (2015). While there is still controversy over how and when *Campylobacter* colonize the ceca, the most common route is believed to be horizontal transmission throughout the flock (Cox et al., 2010; Silva et al., 2011). Vertical transmission has been reported from parent to fertile egg and studies have detected 35% inoculation of the progeny (Clark and Bueschkens, 1985; Chuma et al., 1994; Cox et al., 2010). However, in a study with 60,000 progeny-parent breeders there was no evidence of vertical transmission, and therefore more emphasis is placed on *Campylobacter* colonization occurring through horizontal or environmental transmission (Callicott et al., 2006; Silva et al., 2011). *Campylobacter* can spread through water supplies, insects, litter, rodents, fecal content, and from bird to bird contact (Aarts et al., 1995; Adkin et al., 2006; Horrocks et al., 2009). *Campylobacter* colonization is usually detected at approximately 3 weeks of age and with concentrations rapidly reaching 10^7^ CFU/g (Corry and Atabay, 2001). The ceca, containing up to 10^9^ CFU/g, contains the largest concentration of *Campylobacter* within the avian GIT due to the abundance of nutrients, including amino acids, and the temperature in the avian ceca being approximately 42°C, which is optimal for *Campylobacter* growth (Stern, 2008; Gerwe et al., 2010; Troxell et al., 2015). The ceca are closed pouches between the ileum and the colon (Duke, 1986). This site is an important consideration for food safety as the ceca may rupture during poultry processing leading to contamination of the finished poultry product if not properly handled (Hargis et al., 1995).

### EOs and *Campylobacter* in the Ceca

When examining responses of cecal contents *in vitro*, 20 mM (approximately 0.3%) of cinnamaldehyde, thymol, eugenol, or carvacrol were all independently effective in significantly reducing *Campylobacter* concentrations after 15 s of incubation (Kollanoor-Johny et al., 2010). By 8 h incubation, 10 mM concentrations of cinnamaldehyde, thymol, eugenol, or carvacrol were sufficient in decreasing *C. jejuni* by at least 5-log colony forming units (CFUs)/mL (Kollanoor-Johny et al., 2010). Kurekci et al. (2013) spiked 3 × 10^8^ CFU/ml of *C. jejuni* C338 into 20-day old chicken cecal contents that previously contained no detectable *Campylobacter.* One gram of cecal contents was mixed with 19 mL of an anaerobic media containing 0.05 or 0.025% lemon myrtle oil. The media comprised of MgSO_4_7H_2_O, 0.5 g; CaCl_2_, 0.02 g; K_2_HPO_4_, 0.75 g; NaH_2_PO_4_H_2_O, 0.25 g; yeast extract 1.0 g; resazurin, 1 mg; and cysteine-HCl, 0.5 g per liter of deionized water was kept in an anaerobic chamber (Laanbroek et al., 1977). Cultures were incubated for 48 h at 39°C and plated. While the positive control retained a concentration of 6.11 log CFU/mL, broths containing EOs reduced *Campylobacter* concentrations below the limit of detection (less than 3.3 log CFU/mL).

Caprylic acid, a component of coconut oil and palm kernel oil, significantly reduced *Campylobacter* cecal concentrations when administered in feed at concentrations below 1% (Los Santos et al., 2008, 2009). This was observed in market age and 10-day old broilers and did not affect FCR or BWG. However, the *in vivo Campylobacter* reducing effects of caprylic acid have been demonstrated to be mitigated when applied as caprylate in feed and water (Hermans et al., 2010; Metcalf et al., 2011).

Arsi et al. (2014) investigated the use of thymol, carvacrol, or a combination, as a feed amendment to prevent *C. jejuni* colonization. Ten birds per treatment were inoculated on day 3 with a 5-strain mixture of *C. jejuni* that were previously isolated from chicken ceca and susceptible to ciprofloxacin or fluoroquinolone. Birds were euthanized on day 10. Cecal *Campylobacter* counts were enumerated using *Campylobacter* Line agar (Line, 2001) and confirmed using latex agglutination. Individual strains were not distinguished. Four trials were performed of this experiment with the only difference between trials was the inclusion of a 2% (Trials 1 and 2) or 0.125% treatment group and the use of EO combinations (Trials 3 and 4). While significant reductions of *Campylobacter* in the 0.25% thymol, 2% thymol, 1% carvacrol, and 0.5% thymol and carvacrol treatments existed, these reductions were not observed across all trials and were not detected in birds fed other concentrations. Thymol, at a concentration of 0.25%, reduced *Campylobacter* by 0.6 log CFU/mL cecal contents during only one trial out of four. A 2 log CFU reduction was observed with 2% thymol for only one trial out of two. Additionally, 2 log CFU/mL cecal contents were observed for one trial using 0.5% thymol and carvacrol, but this was not repeated.

*Campylobacter jejuni-*infected broilers in a seeder model that were given feed coated in 0.3% trans-cinnamaldehyde did not exhibit any significant reduction in *Campylobacter* cecal populations after 1 week (Hermans et al., 2011a). In this model, six pens were set up each containing nine chickens with half of the pens receiving cinnamaldehyde. At day 15, three chicks per pen were given 10^8^ CFU/mL of *C. jejuni* KC 40. At day 21, the chickens were euthanized. All ceca within the treatment groups contained *C. jejuni*. When the cecal populations of *Campylobacter* were averaged per pen, significant differences between treatment and control group were not observed. Cinnamaldehyde degrades quickly in the upper GIT of piglets, and this may explain the lack of differences within the populations for the chicken-based studies as well (Michiels et al., 2008). A commercial blend of garlic and cinnamon, Alliin Plus (Orffa, Werkendam, Netherlands), was found to cause a 1 log CFU/g reduction of *Campylobacter* cecal counts 3 days post-infection (day 11) but no significant effects were detected on day 35 or day 42 (Guyard-Nicodeme et al., 2015).

### Combinations of EOs and Other Antimicrobial Compounds

Numerous alternative antimicrobials have been implemented in pre-harvest poultry environments to inhibit foodborne pathogens such as *Campylobacter* (Umaraw et al., 2017). These include bacteriophages, bacteriocins, prebiotics, probiotics, and organic acids (OAs), and EOs (van der Wielen et al., 2000; Edris, 2007; Umaraw et al., 2017). With different mechanisms of action, these remediation techniques may have synergistic potential. As such, this section will review combinations of EOs and other alternative non-EO antimicrobials, notably OAs against *Campylobacter*.

Gracia et al. (2015) evaluated the effectiveness of a 0.03% blend of thymol, eugenol, piperine, and benzoic acid or 0.08% garlic oil. Chickens were inoculated with 0.1 mL of 10^5^ CFU/mL of *C. jejuni* at day 14 by Gracia et al. (2015). On days 21 to 42, birds in treatment groups were administered the EOs benzoic acid blend or garlic oil. In the control groups, *C. jejuni* populations were 7.33 and 7.38 log CFU/g. In the treatment group with the EOs blend, concentrations of *C. jejuni* were 7.66 log CFU/g, and in the garlic oil treatment, *C. jejuni* concentrations were 7.26 log CFU/g. Both these treatments were not able to statistically reduce *Campylobacter* concentrations.

An OA and EO treatment combination was administered to broilers by Thibodeau et al. (2014). This blend contained sorbate, fumarate, and thymol. Sorbate has been demonstrated to disrupt the cell wall of Gram-negative bacteria and lower pH of the GIT, while fumarate indirectly affects intestinal bacteria by lowering the pH of the stomach (Diener et al., 1993; Papatsiros et al., 2013; Dittoe et al., 2018). Because these OAs operate differently than the proposed EO mechanism, they may have synergistic potential, although fumarate has been demonstrated to be metabolized by *C. jejuni* (Hinton, 2006). Feed was amended with 500 ppm of the EOs-OA blend and provided to broilers (Thibodeau et al., 2014). On day 14, these birds were administered 1 mL of inoculum containing 10^5^ CFU/mL of two strains of *Campylobacter* (designated #1 and 2). One, two, and three weeks after inoculation *Campylobacter* concentrations were enumerated in the ceca and on the whole carcass post-processing. This experiment was repeated using a different set of strains (3 and 4) with lower adhesion properties. In the trial with strains 1 and 2, counts of *Campylobacter* were not significantly different when compared to the control. In the trial with strains 3 and 4, cecal populations were significantly higher 3 weeks after inoculation by approximately 1.5 log CFU/g, but carcass rinses were significantly lower by approximately 2 log CFU/mL. This suggests the adhesion properties may impact the efficacy of the EO treatment, however other variations between the strains may be impacting the results. These adhesion properties are necessary for binding to the intestinal cell wall within poultry, which may also be impacted by EOs (Vidanarachchi et al., 2005).

### Impacts on the Intestinal Mucosal Layer and Microbiota

The proposed mechanism of protecting the intestinal mucosa from colonization has been demonstrated by studies involving prebiotics, which are typically oligosaccharides utilized for the protection of the mucosal layer or improvement of the colonization of beneficial bacteria (Lee et al., 2002; Vidanarachchi et al., 2005; Johnson et al., 2015; Roto et al., 2015; Micciche et al., 2018a; Ricke, 2018). The intestinal mucosa consists of the epithelium and lymphoid tissue along with the mucus that is primarily comprised of glycoproteins referred to as mucins (Montagne et al., 2003). Mucins range from 0.5 to 20 Mda in size, and the saccharide component of the avian mucins includes fucose (15.29 ng/μg of mucin), *N*-acetyl-galactosamine (5.3 ng/μg), *N*-acetyl-glucosamine (47.72 ng/μg), galactose (24.67 ng/μg), glucose (5.15 ng/μg), and mannose (15.44 ng/μg) (Bansil and Turner, 2006; Looft et al., 2019). These carbohydrates comprise 80% of the glycoprotein weight and are oligosaccharide chains consisting of 5 to 15 monomers attached to the protein core (Bansil and Turner, 2006). Approximately 60% of the protein core consists of serine, threonine, and proline repeats and this is interspersed with approximately 10% cysteine and connect to the oligosaccharide chains via O-glycosidic bonds (Perez-Vilar and Hill, 1999; Gongqiao et al., 2003; Bansil and Turner, 2006).

Mucus protects the GIT epithelial layer from exposure to the digestive enzymes and corrosive gastric fluids and serves as a matrix for the entrapment of bacteria (Turnberg, 1987; Perez-Vilar and Hill, 1999; Lien et al., 2001). For instance, L-fucose, serine, and cysteine of mucins have been demonstrated to exhibit a positive chemotaxis response on *C. jejuni*, attracting the bacteria to the chemical compound (Hugdahl et al., 1988). *Campylobacter jejuni* have also been found to preferentially attach to avian mucins compared to cow, deer, horses, mice, sheep, pigs, and rat mucins (Naughton et al., 2013). These entrapped bacteria can, in turn, modulate gene expression of epithelial cells, impact lymphoid cells, and affect the overall health of the host by degrading complex oligosaccharides and producing short-chain fatty acids (SCFAs) (Hooper et al., 2001; Guarner and Malagelada, 2003; Sergeant et al., 2014). In chickens, the intestinal microbiota within this mucosal layer is diverse and complex, and in the ceca, the microbiome is primarily colonized by *Firmicutes, Bacteriodetes*, and *Proteobacteria* (Wei et al., 2013).

Detectable *Campylobacter* colonization usually occurs after 14 days of age and growth is often correlated with the presence of other microbiota, although *Campylobacter’s* interaction with the microbiome is poorly characterized (Hermans et al., 2011b; Indikova et al., 2015; Thibodeau et al., 2015). In humans, *C. jejuni* colonization is associated with a decrease of the butyrate producer, *Faecalibacterium*, which suggests these two bacteria may share a similar ecological niche (Hansen et al., 2014; Thibodeau et al., 2015). Lower abundances of *Lactobacillus* and *Corynebacterium* have also been associated with *C. jejuni* colonization, along with higher concentrations of *Streptococcus* and *Ruminococcaceae* (Kaakoush et al., 2014). These findings indicate that there is an interaction between the intestinal microbiota and *C. jejuni* colonization. Therefore, utilizing EOs to alter the mucosal layer, possibly through microbiome modulation, or interfering with *C. jejuni* binding properties, would be beneficial to preventing intestinal colonization and downstream product contamination.

One mechanism for bacterial adhesion to the poultry GIT is through lectin-carbohydrate receptors (Vidanarachchi et al., 2005). Mutants of *C. jejuni* without CadF protein synthesis capabilities are unable to produce a lectin protein that specifically binds to fibronectin (Ziprin et al., 1999; Rubinchik et al., 2012). CadF is a membrane-bound protein that mediates the binding of *Campylobacter* to fibronectin within the intestinal mucosa, which is necessary for *Campylobacter* colonization (Quaroni et al., 1978; Ziprin et al., 1999; Monteville et al., 2003). CadF operates by modulating the level of tyrosine phosphorylation of paxillin, which is a focal adhesion signaling molecule (Konkel et al., 2005). These mutants were unable to colonize the poultry GIT, because of the absence of the carbohydrate binding adhesion protein (Ziprin et al., 1999). Piva and Rossi (1998) postulated that oligosaccharides, such as mannan-oligosaccharides, would bind to enterocyte receptors on pathogenic cell walls which would also prevent colonization (Micciche et al., 2018a). Essential oils have not yet been found to inhibit colonization of *Campylobacter* through preventing lectin-carbohydrate binding.

Similar to prebiotics, such as pectin and oligosaccharides, EOs have been shown to improve the qualities of the mucosal layer of the intestine (Bengmark, 1998; Vidanarachchi et al., 2005; Wang et al., 2016). As with EOs impacting the immune response in layer hens against NCV, prebiotics has been shown to upregulate immune response cells in the intestinal mucosal such as CD4+ and CD8+ (Lourenço et al., 2015). Immune responses by prebiotic supplementation have been extensively reviewed in Hardy et al. (2013). Additionally, mucins, which can subvert the adherence of pathogenic *E. coli* have been upregulated by *Lactobacillus plantarum* 299V and *Lactobacillus rhamnosus* GG (Mack et al., 1999; Hardy et al., 2013). *Lactobacillus* can be promoted by prebiotics and potentially EOs, and this can potentially create a positive feedback loop as mucins have also been demonstrated to improve bacterial growth as they can be up to 90% carbohydrate by weight (Perez-Vilar and Hill, 1999; Manzanilla et al., 2004; Eeckhaut et al., 2008; Emami et al., 2012; Hardy et al., 2013; Yousaf et al., 2017).

The influence by EOs on the intestine has primarily focused on crypt depth and mucosal thickness (Vidanarachchi et al., 2005). Broiler diets supplemented with 100 mg/kg of 5% carvacrol, 3% cinnamaldehyde, and 2% of capsicum oleoresin exhibited an impact on the jejunal mucosa. A higher jejunal wall villi layer was observed along with an increase in thickness of the mucosa layer, which helps prevent bacterial colonization (Jamroz et al., 2006). This is because while the thicker mucosa may potentially entrap more bacteria, a thick mucosal layer may also prevent adhesion to the intestinal villi and subsequent GIT colonization by decreasing the proximity of bacteria to the intestinal binding sites (Turner, 2009). Broilers supplemented with thymol or garlic powder (1 g/kg) in their diet showed similar effects in the intestinal morphology (Demir et al., 2005). However, in pigs, villi length was either decreased or unaffected by supplementation of EOs (Namkung et al., 2004; Nofrarias et al., 2006; Kroismayr et al., 2008). Prevention of bacterial adhesion would be beneficial for inhibiting *Campylobacter* but could have deleterious effects on nutrient absorption due to changes within the GIT microbial community. For instance, SCFA producing bacteria, such as *Lactobacillus* and *Bifidobacterium*, have been shown to have positive impacts on nutrient absorption and overall bird health and antimicrobial strategies that prevent their growth could be detrimental. However, EOs have been reported to improve the colonization of bacteria that are non-pathogenic and may benefit the overall health of the microbiota (Wenk, 2003; Windisch and Kroismayr, 2007).

Growth rate improvement of *Lactobacillus, Bifidobacteria*, and other probiotic bacteria in avian hosts is viewed as a potential mechanism inhibiting avian colonization of foodborne diseases such as *Campylobacter* (Santini et al., 2010). In simulated environments, *Lactobacillus* has been shown to inhibit the colonization of undesired bacteria such as *Campylobacter* (Chang and Chen, 2000). Four strains of *Lactobacillus* (10^4^/ml) and *C. jejuni* (10^6^/mL) were added to a simulated chicken digestive system consisting of pH adjusted veronal buffers. Veronal buffer contains 0.15 mM CaCl_2_, 141 mM NaCl, 0.5 mM MgCl_2_, 0.1% gelatin, 1.8 mM sodium barbital, and 3.1 mM barbituric acid (Sigma-Aldrich; St. Louis, MO, United States) and was subsequently adjusted to a pH of 4.5, 4.4, 2.6, 6.2, and 6.3 to represent the crop, proventriculus, gizzard, small intestine, and large intestine, respectively. A simulated chicken digestive system was also created by adjusting each veronal buffer with 0.1N HCl or with veronal buffer at pH 9.6 to simulate passage within each intestinal organ. Within individually simulated GIT compartments, and the simulated chicken digestive system as a whole, significant *Campylobacter* reductions were observed compared to the control with no addition of *Lactobacillus*. In the gizzard, while a reduction from approximately 6 log CFU/mL to approximately 4 log CFU/mL of *Campylobacter* was observed within 90 min, likely due to the low pH, a 4 log reduction was observed when 6 log CFU/mL of *Lactobacillus* was added. These results might in part be due to the ability for *Lactobacillus* to produce bacteriocins that are inhibitory to *C. jejuni* (Messaoudi et al., 2012). Stern et al. (2006) applied purified bacteriocin OR-7, produced by *Lactobacillus salivarius* NRRL B-30514, to the feed of broilers that were challenged with *C. jejuni*. Compared to controls, 6 log CFU/g cecal content reductions were observed independently of the *C. jejuni* strain utilized.

*Lactobacillus* has also been known to produce SCFAs that can reduce *C. jejuni* populations (Awad et al., 2018). When SCFAs were tested *in vitro*, butyrate was determined to be bactericidal toward *C. jejuni* at a concentration of 12.5 mM, and acetate and propionate were bacteriostatic at 50 mM (Van Deun et al., 2008). *Campylobacter* has been demonstrated to produce acetate as a by-product of serine catabolism and utilize it as an energy source, which could explain the variation between it and butyrate inhibition (Parker et al., 2007; Stahl et al., 2012). This does not explain the variation between propionate and butyrate. While it has been demonstrated that *C. jejuni* can metabolize acetate and the OA lactate, no known pathways have been elucidated for the transport or metabolism of propionate or butyrate (Wright et al., 2009; Thomas et al., 2011; Anand et al., 2016).

*Lactobacillus* and *Enterococcus* strains, which can produce SCFAs, were tested *in vitro* and were also found to be inhibitory to *C. jejuni* (Chaveerach et al., 2004; Allameh et al., 2017). Chaveerach et al. (2004) cultured five *Enterococcus* and five *Lactobacillus* strains from healthy chickens and grew them in Mueller-Hinton broth. The broth was subsequently centrifuged, and the supernatant was separated from the bacteria. The supernatant was neutralized to a pH of 6.2 and treated with pronase and catalase to break down bacteriocins and hydrogen peroxide, respectively. Once the supernatants were confirmed to be free of bacteria, they were applied in a well-diffusion agar assay against 10 individual strains of *C. jejuni*. The supernatant with the highest bactericidal activity came from a *Lactobacillus* strain labeled P93, which produced a zone of inhibition of 9 to 15 mm and was able to impact the growth of all 10 strains of *C. jejuni*. This strain was also grown in co-culture with *C. jejuni* C2150, each at an initial inoculum of 7 log CFU/mL, and after 48 h, *Campylobacter* was not isolated from the culture. *Campylobacter jejuni* C2150 was also grown with *Lactobacillus* strain P104, which did not exhibit any antimicrobial effects in the well-diffusion agar, but by 72 h *Campylobacter* concentrations were approximately 1.5 log CFU/mL lower than the positive control at 7.5 CFU/mL. This study indicates that not only can *Lactobacillus* directly compete for nutrients and colonization niche with *Campylobacter*, but some strains can also employ antimicrobials to reduce their population numbers further.

Plant-derived compounds such as cumin, oregano, and extracts of capsaicin, cinnamaldehyde, and carvacrol have been shown to improve the growth of *Lactobacillus, Bifidobacteria*, and *Enterococcus*, and therefore may indirectly impact pathogen concentrations through competitive exclusion (Kivanç et al., 1991; Jamroz et al., 2003; Manzanilla et al., 2004). Manzanilla et al. (2001) demonstrated how XT, a mixture of carvacrol, cinnamaldehyde, and capsaicin, increased *Lactobacillus* cecal counts in post-weaning pigs when provided in the feed. However, these interactions are complicated as the addition of *C. jejuni* has been attributed to an increase in *Bifidobacterium* (Thibodeau et al., 2015). Further research must be performed to determine if, or which, EOs exhibit antimicrobial qualities against *Campylobacter* that may also simultaneously support GIT bacteria that are antagonistic to *Campylobacter*. While EOs do not appear to operate as substrates for *Lactobacillus* or other probiotics and are therefore not prebiotics, their indirect impacts on the intestinal mucosal layer promote an environment suitable for the growth of “beneficial” bacteria, which in turn, have the potential to reduce *Campylobacter* concentrations. Other studies found that EOs, including cumin, orange oil, and oregano, have an inhibitory effect on *Lactobacillus* spp. (Kivanç et al., 1991; Elgayyar et al., 2001; Delaquis et al., 2002; Chalova et al., 2010). As such, there are limitations for the application of EOs to poultry that must be addressed.

### Potential Limitations and Future Directions for Preharvest EOs Application

There appears to be inconsistency in responses of birds infected by *Campylobacter* when administered EOs compared to *in vitro* responses as well as among individual bird trials. Understanding the underlying factors and potential limitations may give insight into how EOs can be applied in the future. One primary limitation of EOs is that they can be rapidly absorbed in the GIT (Meunier et al., 2006). This rapid absorption has been observed in pigs and humans (Kohlert et al., 2002; Meunier et al., 2006). Absorption by the stomach and small intestine before they can affect cecal concentrations may be occuring in *in vivo* studies (Arsi et al., 2014). To test this potential limitation, intestinal absorption resistant EOs derivatives can be applied. Thymol-β-d-glucopyranoside is more resistant to intestinal absorption than thymol and has been shown to have similar antimicrobial effects *in vitro* (Epps et al., 2015). However, while a 1 log reduction of *Campylobacter* was observed in the crops of market-aged broilers with this absorption-resistant compound, no significant impacts were found in the ceca with either thymol-based treatment (Epps et al., 2015).

Microencapsulation may also be utilized (Calo et al., 2015). Microencapsulation is a process in which liquid particles are surrounded in polymeric compounds (Bansode et al., 2010). Typical water-soluble coating materials can include gelatin, gum arabic, and polyacrylic acid (Jyothi et al., 2012). The intention of microencapsulation with preharvest remediation techniques is to prevent the therapeutic compound from absorption before reaching the target area within the GIT (Van Immerseel et al., 2004). For instance, Pan et al. (2014) found that encapsulated thymol inhibited pathogens more effectively in milk due to enhanced solubility. As such, microencapsulated EOs have been suggested as a methodology for improving the *in vivo* effects of EOs (Calo et al., 2015). Utilizing a feed-based microencapsulated blend of thymol and eugenol, along with propionic and sorbic acids, Grilli et al. (2013) was able to reduce *C. jejuni* in layer hens. During a 42-day trial, birds were provided this blend, labeled CTR, at varying concentrations (0.1, 0.3, 0.5, or 1.0%), and were infected with 10^7^ CFU/mL *C. jejuni* on day 22. *Campylobacter* concentrations were measured by plating cecal contents onto modified Charcoal-Cefoperazone-Deoxycholate Agar (mCCDA). The CTR blend was effective in reducing *C. jejuni* at all concentrations (0.1, 0.3, 0.5, or 1.0%) on days 35 and 43, with 1% CTR exhibiting a 5 log CFU/g reduction at day 42 compared to the control. At day 42 the 0.1% concentration reduced *Campylobacter* populations by 3 log CFU/g. In a second experiment utilizing either 0.1 or 0.3% of this blend, birds were given either the blend before or after the day 22 infection with *Campylobacter*. Statistically significant reductions were still observed within each treatment group, except with 0.1% CTR at day 35, but the reductions were significantly lower for the group given the treatment after infection instead of before. The CTR blend at a concentration of 0.3% given from day 0 to 21 reduced *C. jejuni* by 1.5 log CFU/g at day 35 where 3 log CFU/g reductions were observed when the 0.3% CTR blend was applied on day 22. However, in a cecal loop model study by Hermans et al. (2011a), involving direct injection of the trans-cinnamaldehyde into ceca infected with *C. jejuni* they failed to detect significant reductions in *Campylobacter* concentrations. This indicates that the absorption of the EOs in the upper GIT is not the only reason for variation between *in vivo* and *in vitro* trials (Arsi et al., 2014). Further experiments utilizing microencapsulation of combined EO blends, along with comparisons with their unencapsulated counterparts, is necessary to determine the specific effectiveness of utilizing microencapsulation.

Additionally, the observed reductions using blends of EOs can help reduce the incidence of *Campylobacter* at time of slaughter, which can lead to reductions of human incidences of campylobacteriosis (Rosenquist et al., 2003; Chapman et al., 2016). Furthermore, an investigation into EOs impact on the GIT microbiome may also represent a pertinent future direction. For instance, increasing the concentrations of certain members of the GIT microbiome through EOs may generate a GIT less suitable or more hostile for initial *C. jejuni* colonization (Kaakoush et al., 2014). Once the interactions between *C. jejuni*, the microbiome, and EOs remediation are fully elucidated a more targeted remediation technique may be possible. For instance, specific EOs blends could be designed to modulate the microbiome to prevent *C. jejuni* colonization. While *in vivo* EOs application may not be able to eliminate *Campylobacter* concentrations at the time of slaughter, it does provide a hurdle that the bacteria must overcome before contamination of the final product (Leistner, 2000; Holley and Patel, 2005).

## Antimicrobial Effects of EOs on *Campylobacter* in Poultry Processing

*Campylobacter* contamination on poultry products is one of the more common causes of campylobacteriosis in humans (Keener et al., 2004). Incidence rates in a study of 425 broiler carcasses over 12 months revealed 87.5% of the post-chill carcasses were contaminated with *Campylobacter* in a French slaughterhouse (Hue et al., 2010). In the United States, 52% of post-chill carcasses (*n* = 325) were contaminated with *Campylobacter* with 100% of the carcasses being contaminated pre-chill (Son et al., 2007). The most frequent method for this contamination event is for the GIT to rupture during processing and contaminants to spill onto the carcass (Berrang et al., 2001). If not properly treated during carcass rinses, cross-contamination can occur, especially within chiller tanks (Bashor et al., 2004). As such, to prevent cross-contamination, it is vital that carcass rinses and sprays be applied as appropriate sanitation techniques. Essential oils may serve as an alternative sanitizer for processing washes.

*In vitro* laboratory experiments may have more relevance to post-harvest interventions than pre-harvest mediations due to the complexity of the poultry GIT that cannot easily be modeled in a laboratory environment (White et al., 1997). For instance, in a series of benchtop studies orange oil was found to be inhibitory to *C. jejuni, C. coli, L. monocytogenes, Salmonella*, and *Pseudomonas* (O’Bryan et al., 2008; Nannapaneni et al., 2009; Chalova et al., 2010). Nannapaneni et al. (2009) tested seven orange oil fractions on 3 *Arcobacter* strains and 21 *Campylobacter* strains, including 14 *C. jejuni* strains, four of which were isolated from poultry. When viewed on disk diffusion agar, cold pressed (CP) terpeneless Valencia orange oil produced the largest zones of inhibition (in mm) of *C. jejuni* including those isolated in poultry, whereas other orange oil fractions produced more limited zones of inhibition. Valencia orange oil was also reported to be inhibitory toward *C. coli* and *Arcobacter*. Kurekci et al. (2013) found tea tree oil at a concentration of 0.001% to be inhibitory against two strains of *C. jejuni* on nutrient agar. Wild carrot oil, when used in agar plates as an antimicrobial against *Campylobacter* spp. including a multidrug-resistant strain, *C. jejuni* 99T403, exhibited a minimum inhibitory concentration (MIC) of 125 to 500 μg/mL depending on the species or strain (Rossi et al., 2007). Isoeugenol and E-methyl isoeugenol are extracts of carrot oil and when tested against *C. jejuni* resulted in an MIC of 125 μg/mL (Rossi et al., 2007). Thymol, in a concentration of 0.25 μmol/mL, reduced *C. jejuni* and *C. coli* by 5 logs CFU/mL in Bolton broth from an initial concentration of 7 log CFU/mL (Anderson et al., 2009; Carocho et al., 2014). An EO extract from *Origanum minutiflorum*, composed primarily of carvacrol and *p-*cymene, has been shown to be effective in inhibiting *C. jejuni* in concentrations as low as 12.5 μg/mL on Mueller-Hinton agar (Aslim and Yucel, 2008). However, these MICs were strain-specific, as of the 12 *C. jejuni* strains tested, only *C. jejuni* 118d was inhibited at the concentration of 12.5 μg/mL, while *C. jejuni* 113k, 7d, and 9a required 700 μg/mL to be inhibited (Aslim and Yucel, 2008). Cinnamon, clove, thyme, and bay leaf oils were found to be bacteriostatic at a concentration of 0.075% against *C. jejuni, S.* Enteritidis*, E. coli, Staphylococcus aureus*, and *L. monocytogenes* when tested in tryptic soy broth (TSB) (Smith-Palmer et al., 1998).

Other studies reported similar inhibitory and bacteriostatic effects of oregano, eucalyptus, marigold, ginger, jasmine, cedarwood, carrot, mugwort, bergamot, and other EOs, against *Campylobacter* and other foodborne pathogens (Friedman et al., 2002; Moreira et al., 2005; Fisher and Phillips, 2006; Thanissery et al., 2014). The MIC of EOs on other foodborne pathogens were collected and reviewed in Hyldgaard et al. (2012). Friedman et al. (2002) tested 119 EOs against *C. jejuni, E. coli* O157:H7*, L. monocytogenes*, and *S. enterica* in PBS for 60 min at 37°C (42°C for *C. jejuni*) followed by plating on appropriate media for each species, including an iron-supplemented Brucella agar for *C. jejuni.* The EO concentration that resulted in the bactericidal activity of a 50% CFU decrease relative to the control was determined for each EO bacterial species combination. Marigold taegetes (0.003%), ginger root (0.005%), jasmine (0.006%), patchouli (0.007%), and gardenia (0.007%) were the most effective oils against *C. jejuni* RM1221 followed by cedarwood (0.0075%), carrot seed (0.0078%), celery seed (0.0085%), mugwort (0.009%), spikenard (0.009%), and orange bitter oils (0.009%). In this study, 39 EOs were tested against all four foodborne pathogens. When their bactericidal activities were averaged, the five most effective EOs were cinnamaldehyde (0.03%), thymol (0.05%), Spanish oregano (0.05%), carvacrol (0.06%), and *Oregano origanum* (0.06%). Of these five EOs, *C. jejuni* was impacted the most by the lowest percent concentration compared to the other tested foodborne pathogens.

This suggests that not only may *C. jejuni* be a prime target for EO remediation, but EO that have not been extensively tested may be more optimal candidates to utilize, such as marigold and jasmine. Disk diffusion methods indicated that lemon, sweet orange and bergamot were effective against *L. monocytogenes, S. aureus, B. cereus, E. coli* O157:H7, and *C. jejuni* (Fisher and Phillips, 2006). Each oil was added to a 2 cm disk and then placed on agar plates in the presence of the bacteria of interest, with *C. jejuni* SR 117 being plated on *Campylobacter* agar base with 5% horse blood and incubated at 42°C. No zones of inhibition were detected for *C. jejuni* when orange or citral was added. Bergamot produced a zone of inhibition of 23 mm, and lemon produced a zone of inhibition of 18 mm, which were the smallest zones compared to the other tested bacteria. The zone of inhibition for linalool was greater than 90 mm. The MIC was also determined for bergamot (greater than 4%), lemon (greater than 4%), and linalool (0.06%). While no zone of inhibition was visually detected for orange oil by Fisher and Phillips (2006), when orange oil was supplemented with thyme, the combination EOs demonstrated antimicrobial activity against *Campylobacter* (Thanissery et al., 2014). Similar to Fisher and Phillips (2006), a disk diffusion test was performed using thyme, orange, rosemary, clove, and a 1:1 ratio of thyme and orange oil. This was tested on *C. jejuni* 11601 MD, *C. jejuni* RM1221, and *C. coli* RM2228 along with a cocktail of the three strains. No visible growth on disk diffusion assay across all strains and in the cocktail was visualized when exposed to thyme or clove. For the cocktail, orange oil, rosemary, and the combination of thyme and orange oil produced zones of inhibition of 17, 11, and 20 mm, respectively. A macro-broth dilution assay was also performed using Mueller Hinton broth to determine the MIC and minimum bactericidal concentration (MBC) of these oils. However, the exact MIC and MBC was not determined as 0.0008% concentrations of the oils were sufficient for inhibiting bacterial growth.

Synergistic effects have also been detected (Nguefack et al., 2012). For instance, a combination of oregano and thyme or oregano and cinnamaldehyde required 80% less EOs to produce the same inhibitory effects in nutrient broth with a *Campylobacter* growth supplement (Navarro et al., 2015). Thymol and geraniol reduced *Clostridium difficile* in feces at 500 ppm, which was five times the concentration utilized for a significant reduction in pure cultures. When 0.16 mg/mL of rosemary oil, consisting of carnosic acid, carnosol, and rosmarinic acid was applied in the laboratory, a 2 log CFU/mL reduction in *C. jejuni* was observed in Mueller-Hinton broth when the initial concentration was at 7 log CFU/mL (Klancnik et al., 2009; Piskernik et al., 2011). However, four times the concentration was needed to achieve the same reduction in chicken meat juice, isolated from thawed previously frozen carcasses, unless supplemented with nisin, a bacteriocin (Piskernik et al., 2011). While the complete composition was not provided in Piskernik et al. (2011), it was suggested that lipids and proteins within the juice matrix might have partly inhibited the EO therapy. Burt (2004) suggested that the decreased EO effectiveness in the food matrix compared to the broth experiment may be because in an oil-in-water emulsion that allows the bacteria to grow as films and in colonies, which can shield interior cells from therapeutics. As a consequence, while *in vitro* laboratory models are important, to elucidate the potential impact of EOs, poultry product matrices as well as processing environments must be considered.

The use of EOs in the post-harvest environment has focused on their bactericidal activity within carcasses washes and finished products (Calo et al., 2015; Dima and Dima, 2015). A 0.5% 50:50 mixture of thyme oil and orange oil was used in a marinade for chicken wings dip-inoculated with 10^7^ CFU/mL of a nalidixic acid resistant strain of *C. coli* (Thanissery and Smith, 2014). While cross-contamination events were observed via the marinade, *C. coli* concentrations on treated wings were reduced by 3.0 log CFU/mL, as determined through plating of rinsates. Skinless chicken breasts experimentally infected with 5 × 10^5^ CFU/g of *C. jejuni* were subjected to stinkwort (0.2%; *Inula graveolens*), bay leaf (0.6%; *Laurus nobilis*), mastic tree (0.6%; *Pistacia lentiscus*), and winter savory (0.6%; *Atureja gontana*) (Djenane et al., 2012). After 8 days of refrigerated storage under microaerophilic conditions, greater than 5 log CFU/g reductions of *C. jejuni* were observed compared to the control inoculated with 5 × 10^5^ CFU/g. Sensory analysis indicated these EOs improved or did not impact the odor of the refrigerated samples according to a six member trained panel. Within 60 s, 0.06% linalool oil reduced *C. jejuni* on 2 cm × 2 cm pieces of cabbage leaf and chicken skin by greater than 5 log CFU and 2 log CFU, respectively (Fisher and Phillips, 2006). Cold pressed Valencia orange oil has also been shown to reduce *C. jejuni* UAF 244 on retail chicken thighs and legs (Nannapaneni et al., 2009). Chicken thighs and legs were dipped in a 0.8% saline solution containing 10^6^ CFU/mL *C. jejuni* UAF 244 for 5 min and then submerged for 2 min in 20% (v/v) Valencia orange oil or 20% (v/v) limonene. The samples were then rinsed and plated. Across both types of chicken pieces, 1.5 to 2 log CFU/mL reductions were observed, compared to the control. Treatments with limonene resulted in reductions without detectable recovery of viable bacterial cells, although the limit of detection was not provided by the authors. Moreover, while taste panels have found concentrations of orange oil up to 0.1% to be acceptable in milk, chicken patties and marinades, a 20% part dip has not been investigated for impacts on sensory characteristics (Jo et al., 2004; Fisher and Phillips, 2008; Rimini et al., 2014).

Other factors may confound EO efficacy toward *Campylobacter* in poultry products. For example, one major concern for poultry industries is the ability for pathogens to form biofilms (Srey et al., 2013). Biofilms are bacterial communities within a polysaccharide matrix that can readily form and attach on processing surfaces (Costerton, 1995; Donlan and Costerton, 2002). Biofilms can be difficult to remove by antimicrobials and processing sanitizers such as chlorine and peracetic acid, which are commonly used in the industry (Frank et al., 2003; Ryu and Beuchat, 2005; Scher et al., 2005; Deborde and Von Gunten, 2008). *Campylobacter* is known to form biofilms on stainless steel, polystyrene, and glass (Gunther and Chen, 2009). Coriander oil and its antimicrobial component linalool were found to affect biofilm formation of *Campylobacter* planktonic cells and pre-established biofilms (Duarte et al., 2016). When coriander and linalool were used at 2 μg/mL (approximately four times the MIC) *C. jejuni* and *C. coli* biofilms were reduced in size by 70 to 80% after 48-h incubation in a crystal violet assay (Duarte et al., 2015, 2016). Even when using half the MIC, coriander, and linalool reduced the biofilm by 20 to 25% (Duarte et al., 2016). Results of biofilm inhibition varied more wildly from planktonic cells, but all concentrations (0.025 to 2 μg/mL) successfully inhibited biofilm growth compared to the control, with linalool reducing some biofilm formation between 10 and 20% of the control (Duarte et al., 2016). Thyme, oregano, and cinnamon EOs, when used at concentrations below the MIC, were also found effective against biofilms of *Acinetobacter, Sphingomonas*, and *Stenotrophomonas* spp. that were isolated from biofilms within the food industry (Szczepanski and Lipski, 2014). *Sphingomonas* biofilms were reduced by 50% by thyme oil at a concentration of 0.001% where the MIC was 0.008% (Szczepanski and Lipski, 2014). Similar results were found using thyme and balsam on *Pseudomonas* and *S. aureus* biofilms (Kavanaugh and Ribbeck, 2012; Kerekes et al., 2015).

## Conclusions

The effectiveness of EOs in poultry has not been clearly defined yet. Product advantages have been noted in several studies, but studies also exist that display no impact on FCR or BWG. Other advantages of EOs include the potentially improved flavor of the carcass, antioxidant capacity, and improved feed digestibility. There is also evidence that EOs can be utilized *in vitro* to impact pathogen concentrations, including *Campylobacter*. However, this depends largely on the EO utilized as the mechanism(s) of action are not well-defined. With over 300 commercially available EOs, precisely elucidating the underlying mechanisms may prove difficult (Bajpai et al., 2012). Less information is available regarding the mechanistic role of EOs used *in vivo*. Their potential ability to improve amino acid absorption in the ileum may allow for generating a GIT environment unfavorable to *Campylobacter* in the ceca due to diminished substrate availability, which is further downstream. To fully elucidate the impact EOs have on *Campylobacter* concentrations *in vivo*, further research on the mechanism(s) and effects of EOs must be performed.

More targeted delivery of EOs to certain sites in the avian GIT may be warranted as well. Microencapsulation may help to stabilize the chemical activity of the EO until it reaches its target site in the GIT, thus ensuring less variability. Microencapsulation also holds promise in addressing the intestinal absorption of an antimicrobial. However, investigations into the duration the encapsulated EOs remains in the GIT need to be performed to see when the remediation should be administered. In post-harvest settings, further studies should be performed involving the addition of EOs to sprays, washes, or within the chiller tank.

To determine the specific antimicrobial effects of EOs, the mechanism(s) must be elucidated. This is essential because, with a wide variety of EOs, there may be multiple mechanisms at work and thus synergistic potential which cannot be determined without proper identification of the mode of inhibitory action for each individual EO. Toward this effort, molecular approaches such as transcriptomics and proteomics may be employed to determine which pathways EOs inhibit will lead to further understanding of their impact on *Campylobacter.* While there have been suggestions to utilize EOs as a hurdle technology in poultry production pre- and post-harvest, mechanisms of action against *Campylobacter* and the optimal GIT locations and processing steps must first be established before any practical recommendations can be given.

## Author Contributions

All authors listed have made a substantial, direct and intellectual contribution to the work, and approved it for publication.

## Conflict of Interest Statement

The authors declare that the research was conducted in the absence of any commercial or financial relationships that could be construed as a potential conflict of interest.

## References

[B1] Aarts H. J. Van Lith L. A. Jacobs-Reitsma W. F. (1995). Discrepancy between Penner serotyping and polymerase chain reaction fingerprinting of *Campylobacter* isolated from poultry and other animal sources. *Lett. Appl. Microbiol.* 20 371–374. 10.1111/j.1472-765x.1995.tb01324.x 7786504

[B2] Adkin A. Hartnett E. Jordan L. Newell D. Davidson H. (2006). Use of systematic review to assist the development of *Campylobacter* control strategies in broilers. *J. Appl. Microbiol.* 100 306–315. 10.1111/j.1365-2672.2005.02781.x 16430507

[B3] Alcicek A. Bozkurt M. Çabuk M. (2004). The effect of a mixture of herbal essential oils, an organic acid or a probiotic on broiler performance. *S. Afr. J. Anim. Sci.* 34 217–222.

[B4] Allameh S. K. Ringø E. Yusoff F. M. Daud H. M. Ideris A. (2017). Dietary supplement of *Enterococcus faecalis* on digestive enzyme activities, short chain fatty acid production, immune system response and disease resistance of Javanese carp (*Puntius gonionotus*, Bleeker 1850). *Aquac. Nutr.* 23 331–338. 10.1111/anu.12397

[B5] Allen K. J. Griffiths M. W. (2001). Effect of environmental and chemotactic stimuli on the activity of the *Campylobacter jejuni flaA* sigma (28) promoter. *FEMS Microbiol. Lett.* 205 43–48. 10.1016/s0378-1097(01)00444-x 11728714

[B6] Amerah A. M. Mathis G. Hofacre C. L. (2012). Effect of xylanase and a blend of essential oils on performance and *Salmonella* colonization of broiler chickens challenged with *Salmonella* Heidelberg. *Poult. Sci.* 91 943–947. 10.3382/ps.2011-01922 22399734

[B7] Anand S. Kaur H. Mande S. S. (2016). Comparative *in silico* analysis of butyrate production pathways in gut commensals and pathogens. *Front. Microbiol.* 7:1945. 10.3389/fmicb.2016.01945 27994578 PMC5133246

[B8] Anderson R. C. Krueger N. A. Byrd J. A. Harvey R. B. Callaway T. R. Edrington T. S. (2009). Effects of thymol and diphenyliodonium chloride against *Campylobacter* spp. during pure and mixed culture in vitro. *J. Appl. Microbiol.* 107 1258–1268. 10.1111/j.1365-2672.2009.04308.x 19486394

[B9] Ansari-Lari M. Hosseinzadeh S. Shekarforoush S. S. Abdollahi M. Berizi E. (2011). Prevalence and risk factors associated with *Campylobacter* infections in broiler flocks in Shiraz, southern Iran. *Int. J. Food Microbiol.* 144 475–479. 10.1016/j.ijfoodmicro.2010.11.003 21131089

[B10] Arsi K. Donoghue A. M. Venkitanarayanan K. Kollanoor-Johny A. Fanatico A. C. Blore P. J. (2014). The efficacy of the natural plant extracts, thymol and carvacrol against *Campylobacter* colonization in broiler chickens. *J. Food Saf.* 34 321–325. 10.1111/jfs.12129

[B11] Aslim B. Yucel N. (2008). In vitro antimicrobial activity of essential oil from endemic *Origanum minutiflorum* on ciprofloxacin-resistant *Campylobacter* spp. *Food Chem.* 107 602–606. 10.1016/j.foodchem.2007.08.048

[B12] Austgulen L. T. Solheim E. Schelin R. R. (1987). Metabolism in rats of p-cymene derivates: carvacrol and thymol. *Pharmacol. Toxicol.* 61 98–102. 10.1111/j.1600-0773.1987.tb01783.x 2959918

[B13] Awad W. A. Hess C. Hess M. (2018). Re-thinking the chicken–*Campylobacter jejuni* interaction: a review. *Avian Pathol.* 47 352–363. 10.1080/03079457.2018.1475724 29764197

[B14] Bajpai V. K. Baek K.-H. Kang S. C. (2012). Control of *Salmonella* in foods by using essential oils: a review. *Food Res. Int.* 45 722–734. 10.1016/j.foodres.2011.04.052

[B15] Bakkali F. Averbeck S. Averbeck D. Idaomar M. (2008). Biological effects of essential oils: a review. *Food Chem. Toxicol.* 46 446–475. 17996351 10.1016/j.fct.2007.09.106

[B16] Bansil R. Turner B. S. (2006). Mucin structure, aggregation, physiological functions and biomedical applications. *Curr. Opin. Colloid Interface Sci.* 11 164–170. 10.1016/j.cocis.2005.11.001

[B17] Bansode S. S. Banarjee S. K. Gaikwad D. D. Jadhav S. L. Thorat R. M. (2010). Microencapsulation: a review. *Int. J. Pharm. Sci. Rev. Res.* 1 38–43.

[B18] Baratta M. T. Dorman H. D. Deans S. G. Figueiredo A. C. Barroso J. G. Ruberto G. (1998). Antimicrobial and antioxidant properties of some commercial essential oils. *Flavour Fragr. J.* 13 235–244. 10.1002/(sici)1099-1026(1998070)13:4<235::aid-ffj733>3.3.co;2-k

[B19] Bashor M. P. Curtis P. A. Keener K. M. Sheldon B. W. Kathariou S. Osborne J. A. (2004). Effects of carcass washers on *Campylobacter* contamination in large broiler processing plants. *Poult. Sci.* 83 1232–1239. 10.1093/ps/83.7.1232 15285518

[B20] Basmacioğlu Malayoğlu H. Baysal Ş. Misirlioğlu Z. Polat M. Yilmaz H. Turan N. (2010). Effects of oregano essential oil with or without feed enzymes on growth performance, digestive enzyme, nutrient digestibility, lipid metabolism and immune response of broilers fed on wheat–soybean meal diets. *Br. Poult. Sci.* 51 67–80. 10.1080/00071660903573702 20390571

[B21] Batz M. B. Hoffmann S. Morris J. G. Jr. (2012). Ranking the disease burden of 14 pathogens in food sources in the United States using attribution data from outbreak investigations and expert elicitation. *J. Food Prot.* 75 1278–1291. 10.4315/0362-028X.JFP-11-418 22980012

[B22] Baurhoo B. Ruiz-Feria C. A. Zhao X. (2008). Purified lignin: nutritional and health impacts on farm animals—A review. *Anim. Feed Sci. Technol.* 144 175–184. 10.1016/j.anifeedsci.2007.10.016

[B23] Beery J. T. Hugdahl M. B. Doyle M. P. (1988). Colonization of gastrointestinal tracts of chicks by *Campylobacter jejuni*. *Appl. Environ. Microbiol.* 54 2365–2370. 3060015 10.1128/aem.54.10.2365-2370.1988 PMC204261

[B24] Benavides S. Villalobos-Carvajal R. Reyes J. E. (2012). Physical, mechanical and antibacterial properties of alginate film: effect of the crosslinking degree and oregano essential oil concentration. *J. Food Eng.* 110 232–239. 10.1016/j.jfoodeng.2011.05.023

[B25] Bengmark S. (1998). Immunonutrition: role of biosurfactants, fiber, and probiotic bacteria. *Nutrition* 14 585–594. 10.1016/s0899-9007(98)00030-6 9684261

[B26] Berrang M. E. Buhr R. J. Cason J. A. Dickens J. A. (2001). Broiler carcass contamination with *Campylobacter* from feces during defeathering. *J. Food Prot.* 64 2063–2066. 10.4315/0362-028x-64.12.2063 11770639

[B27] Bolton D. J. (2015). *Campylobacter* virulence and survival factors. *Food Microbiol.* 48 99–108. 10.1016/j.fm.2014.11.017 25790997

[B28] Bozkurt M. Küçükyilmaz K. Catli A. U. Çınar M. Bintaş E. Çöven F. (2012). Performance, egg quality, and immune response of laying hens fed diets supplemented with mannan-oligosaccharide or an essential oil mixture under moderate and hot environmental conditions. *Poult. Sci.* 91 1379–1386. 10.3382/ps.2011-02023 22582296

[B29] Brenes A. Roura E. (2010). Essential oils in poultry nutrition: main effects and modes of action. *Anim. Feed Sci. Technol.* 158 1–14. 10.1016/j.anifeedsci.2010.03.007

[B30] Buchbauer G. Jirovetz L. Jäger W. (1991). Aromatherapy: evidence for sedative effects of the essential oil of lavender after inhalation. *Z. Naturforsch. C* 46 1067–1072. 10.1515/znc-1991-11-1223 1817516

[B31] Burt S. (2004). Essential oils: their antibacterial properties and potential applications in foods—a review. *Int. J. Food Microbiol.* 94 223–253. 10.1016/j.ijfoodmicro.2004.03.022 15246235

[B32] Cabuk M. Bozkurt M. Alcicek A. Akbaþ Y. Küçükyýlmaz K. (2006). Effect of a herbal essential oil mixture on growth and internal organ weight of broilers from young and old breeder flocks. *S. Afr. J. Anim. Sci.* 36 135–141.

[B33] Callaway T. R. Carroll J. A. Arthington J. D. Edrington T. S. Anderson R. C. Ricke S. C. (2011). “Citrus products and their use against bacteria: potential health and cost benefits,” in *Nutrients, Dietary Supplements, and Nutriceuticals*, eds Watson R. R. Gerald J. K. Preedy V. R. (New York, NY: Humana Press), 277–286. 10.1007/978-1-60761-308-4_17

[B34] Callicott K. A. Friðriksdóttir V. Reiersen J. Lowman R. Bisaillon J. R. Gunnarsson E. (2006). Lack of evidence for vertical transmission of *Campylobacter* spp. in chickens. *Appl. Environ. Microbiol.* 72 5794–5798. 10.1128/aem.02991-05 16957196 PMC1563688

[B35] Calo J. R. Crandall P. G. O’Bryan C. A. Ricke S. C. (2015). Essential oils as antimicrobials in food systems–A review. *Food Control* 54 111–119. 10.1016/j.foodcont.2014.12.040

[B36] Carocho M. Barreiro M. F. Morales P. Ferreira I. C. (2014). Adding molecules to food, pros and cons: a review on synthetic and natural food additives. *Compr. Rev. Food Sci. Food Saf.* 13 377–399. 10.1111/1541-4337.12065 33412697

[B37] Carson C. F. Mee B. J. Riley T. V. (2002). Mechanism of action of *Melaleuca alternifolia* (tea tree) oil on *Staphylococcus aureus* determined by time-kill, lysis, leakage, and salt tolerance assays and electron microscopy. *Antimicrob. Agents Chemother.* 46 1914–1920. 10.1128/aac.46.6.1914-1920.2002 12019108 PMC127210

[B38] Caspi R. Billington R. Ferrer L. Foerster H. Fulcher C. A. Keseler I. M. (2015). The MetaCyc database of metabolic pathways and enzymes and the BioCyc collection of pathway/genome databases. *Nucleic Acids Res.* 44 D471–D480. 10.1093/nar/gkv1164 26527732 PMC4702838

[B39] Centers for Disease Control and Prevention [CDC] (2017a). *Foodborne Diseases Active Surveillance Network (FoodNet): FoodNet 2015 Surveillance Report (Final Data).* Atlanta, GA: U.S. Department of Health and Human Services.

[B40] Centers for Disease Control and Prevention [CDC] (2017b). *Reports of Selected Campylobacter Outbreak Investigations.*

[B41] Centers for Disease Control and Prevention [CDC] (2018). *Campylobacter (Campylobacteriosis).*

[B42] Cervantes H. M. (2015). Antibiotic-free poultry production: is it sustainable? *J. Appl. Poult. Res.* 24 91–97. 10.3382/japr/pfv006

[B43] Chalova V. I. Crandall P. G. Ricke S. C. (2010). Microbial inhibitory and radical scavenging activities of cold-pressed terpeneless Valencia orange (*Citrus sinensis*) oil in different dispersing agents. *J. Sci. Food Agric.* 90 870–876. 10.1002/jsfa.3897 20355124

[B44] Chang M. H. Chen T. C. (2000). Reduction of *Campylobacter jejuni* in a simulated chicken digestive tract by lactobacilli cultures. *J. Food Prot.* 63 1594–1597. 10.4315/0362-028x-63.11.1594 11079707

[B45] Chapman B. Otten A. Fazil A. Ernst N. Smith B. A. (2016). A review of quantitative microbial risk assessment and consumer process models for *Campylobacter* in broiler chickens. *Microb. Risk Anal.* 2 3–15. 10.1016/j.mran.2016.07.001

[B46] Chaveerach P. Lipman L. J. A. Van Knapen F. (2004). Antagonistic activities of several bacteria on in vitro growth of 10 strains of *Campylobacter jejuni*/*coli*. *Int. J. Food Microbiol.* 90 43–50. 10.1016/s0168-1605(03)00170-3 14672829

[B47] Chouliara E. Karatapanis A. Savvaidis I. N. Kontominas M. G. (2007). Combined effect of oregano essential oil and modified atmosphere packaging on shelf-life extension of fresh chicken breast meat, stored at 4 C. *Food Microbiol.* 24 607–617. 10.1016/j.fm.2006.12.005 17418312

[B48] Chuma T. Yamada T. Yano K. Okamoto K. Yugi H. (1994). A survey of *Campylobacter jejuni* in broilers from assignment to slaughter using DNA-DNA hybridization. *J. Vet. Med. Sci.* 56 697–700. 10.1292/jvms.56.697 7999894

[B49] Clark A. G. Bueschkens D. H. (1985). Laboratory infection of chicken eggs with *Campylobacter jejuni* by using temperature or pressure differentials. *Appl. Environ. Microbiol.* 49 1467–1471. 4015086 10.1128/aem.49.6.1467-1471.1985 PMC241748

[B50] Connerton P. L. Richards P. J. Lafontaine G. M. O’Kane P. M. Ghaffar N. Cummings N. J. (2018). The effect of the timing of exposure to *Campylobacter jejuni* on the gut microbiome and inflammatory responses of broiler chickens. *Microbiome* 6:88. 10.1186/s40168-018-0477-5 29753324 PMC5948730

[B51] Corcionivoschi N. Alvarez L. A. Sharp T. H. Strengert M. Alemka A. Mantell J. (2012). Mucosal reactive oxygen species decrease virulence by disrupting *Campylobacter jejuni* phosphotyrosine signaling. *Cell Host Microbe* 12 47–59. 10.1016/j.chom.2012.05.018 22817987 PMC3749511

[B52] Corry J. E. L. Atabay H. I. (2001). Poultry as a source of *Campylobacter* and related organisms. *J. Appl. Microbiol.* 90 96S–114S. 11422565 10.1046/j.1365-2672.2001.01358.x

[B53] Cosentino S. Tuberoso C. I. G. Pisano B. Satta M. L. Mascia V. Arzedi E. (1999). *In-vitro* antimicrobial activity and chemical composition of Sardinian *Thymus* essential oils. *Lett. Appl. Microbiol.* 29 130–135. 10.1046/j.1472-765x.1999.00605.x 10499301

[B54] Costerton J. W. (1995). Overview of microbial biofilms. *J. Ind. Microbiol.* 15 137–140. 10.1007/bf01569816 8519468

[B55] Cox N. A. Richardson L. J. Buhr R. J. Fedorka-Cray P. J. (2010). *Campylobacter can Remain in Various Organs – WorldPoultry.net.*

[B56] Dang S. Sun L. Huang Y. Lu F. Liu Y. Gong H. (2010). Structure of a fucose transporter in an outward-open conformation. *Nature* 467 734–738. 10.1038/nature09406 20877283

[B57] Davis L. DiRita V. (2017). Growth and laboratory maintenance of *Campylobacter jejuni*. *Curr. Protoc. Microbiol.* 10 8A.1.1–8A.1.7. 10.1002/9780471729259.mc08a01s10 18729058

[B58] De Cesare A. Sheldon B. W. Smith K. S. Jykus L. A. (2003). Survival and persistence of *Campylobacter* and *Salmonella* species under varying organic loads on food contact surfaces. *J. Food Prot.* 66 1587–1594. 10.4315/0362-028x-66.9.1587 14503710

[B59] De Sousa J. P. de Araújo Torres R. de Azerêdo G. A. Figueiredo R. C. da Silva Vasconcelos M. A. de Souza E. L. (2012). Carvacrol and 1, 8-cineole alone or in combination at sublethal concentrations induce changes in the cell morphology and membrane permeability of *Pseudomonas fluorescens* in a vegetable-based broth. *Int. J. Food Microbiol.* 158 9–13. 10.1016/j.ijfoodmicro.2012.06.008 22795513

[B60] Deborde M. Von Gunten U. R. S. (2008). Reactions of chlorine with inorganic and organic compounds during water treatment—kinetics and mechanisms: a critical review. *Water Res.* 42 13–51. 10.1016/j.watres.2007.07.025 17915284

[B61] Debruyne L. Gevers D. Vandamme P. (2008). “Taxonomy of the Family Campylobacteraceae,” in *Campylobacter*, 3rd Edn, eds Nachamkin I. Szymanski C. Blaser M. (Washington, DC: ASM Press), 3–25. 10.1128/9781555815554.ch1

[B62] Delaquis P. J. Stanich K. Girard B. Mazza G. (2002). Antimicrobial activity of individual and mixed fractions of dill, cilantro, coriander and eucalyptus essential oils. *Int. J. Food Microbiol.* 74 101–109. 10.1016/s0168-1605(01)00734-6 11929164

[B63] Demir E. Sarica S. Ozcan M. A. Suicmez M. (2005). The use of natural feed additives as alternatives to an antibiotic growth promoter in broiler diets. *Arch. Geflugelkunde* 69 110–116. 19579918

[B64] Denli M. Okan F. Uluocak A. N. (2004). Effect of dietary supplementation of herb essential oils on the growth performance, carcass and intestinal characteristics of quail (*Coturnix coturnix japonica*). *S. Afr. J. Anim. Sci.* 34 174–179.

[B65] Di Pasqua R. Mamone G. Ferranti P. Ercolini D. Mauriello G. (2010). Changes in the proteome of *Salmonella enterica* serovar Thompson as stress adaptation to sublethal concentrations of thymol. *Proteomics* 10 1040–1049. 10.1002/pmic.200900568 20049861

[B66] Diaz-Sanchez S. D’Souza D. Biswas D. Hanning I. (2015). Botanical alternatives to antibiotics for use in organic poultry production. *Poult. Sci.* 94 1419–1430. 10.3382/ps/pev014 25743421

[B67] Diener M. Helmle-Kolb C. Murer H. Scharrer E. (1993). Effect of short-chain fatty acids on cell volume and intracellular pH in rat distal colon. *Pflügers Arch.* 424 216–223. 10.1007/bf00384345 8414909

[B68] Dima C. Dima S. (2015). Essential oils in foods: extraction, stabilization, and toxicity. *Curr. Opin. Food Sci.* 5 29–35. 10.1016/j.tplants.2016.10.005 27789158

[B69] Dittoe D. K. Ricke S. C. Kiess A. S. (2018). Organic acids and potential for modifying the avian gastrointestinal tract and reducing pathogens and disease. *Front. Vet. Sci.* 5:216. 10.3389/fvets.2018.00216 30238011 PMC6136276

[B70] Díaz E. Ferrández A. Prieto M. A. García J. L. (2001). Biodegradation of aromatic compounds by *Escherichia coli*. *Microbiol. Mol. Biol. Rev.* 65 523–569. 10.1128/mmbr.65.4.523-569.2001 11729263 PMC99040

[B71] Djenane D. Yangueela J. Gomez D. Roncales P. (2012). perspectives on the use of essential oils as antimicrobials against *Campylobacter jejuni* CECT 7572 in retail chicken meats packaged in microaerobic atmosphere. *J. Food Saf.* 32 37–47. 10.1111/j.1745-4565.2011.00342.x

[B72] Donlan R. M. Costerton J. W. (2002). Biofilms: survival mechanisms of clinically relevant microorganisms. *Clin. Microbiol. Rev.* 15 167–193. 10.1128/cmr.15.2.167-193.2002 11932229 PMC118068

[B73] Donsi F. Annunziata M. Sessa M. Ferrari G. (2011). Nanoencapsulation of essential oils to enhance their antimicrobial activity in foods. *LWT Food Sci. Technol.* 44 1908–1914. 10.1016/j.foodchem.2018.11.078 30583392

[B74] Duarte A. Luís Â. Oleastro M. Domingues F. C. (2016). Antioxidant properties of coriander essential oil and linalool and their potential to control *Campylobacter* spp. *Food Control* 61 115–122. 10.1016/j.foodcont.2015.09.033

[B75] Duarte A. Martinho A. Luís A. Figueiras A. Oleastro M. Domingues F. C. (2015). Resveratrol encapsulation with methyl-b-cyclodextrin for antibacterial and antioxidant delivery applications. *LWT Food Sci. Technol.* 63 1254–1260. 10.1016/j.lwt.2015.04.004

[B76] Duke G. E. (1986). “Alimentary canal: anatomy, regulation of feed, and motility,” in *Avian Physiology*, ed. Sturkie P. D. (New York, NY: Springer-Verlag), 269–302.

[B77] Dunkley K. D. Dunkley C. S. Njongmeta N. L. Callaway T. R. Hume M. E. Kubena L. F. (2007). Comparison of *in vitro* fermentation and molecular microbial profiles of high-fiber feed substrates incubated with chicken cecal inocula. *Poult. Sci.* 86 801–810. 10.1093/ps/86.5.801 17435012

[B78] Economou K. D. Oreopoulou V. Thomopoulos C. D. (1991). Antioxidant activity of some plant extracts of the family Labiatae. *J. Am. Oil Chem. Soc.* 68 109–113. 10.1007/bf02662329

[B79] Edris A. E. (2007). Pharmaceutical and therapeutic potentials of essential oils and their individual volatile constituents: a review. *Phytother. Res.* 21 308–323. 10.1002/ptr.2072 17199238

[B80] Eeckhaut V. Van Immerseel F. Dewulf J. Pasmans F. Haesebrouck F. Ducatelle R. (2008). Arabinoxylooligosaccharides from wheat bran inhibit *Salmonella* colonization in broiler chickens. *Poult. Sci.* 87 2329–2334. 10.3382/ps.2008-00193 18931184

[B81] Elgayyar M. Draughon F. A. Golden D. A. Mount J. R. (2001). Antimicrobial activity of essential oils from plants against selected pathogenic and saprophytic microorganisms. *J. Food Prot.* 64 1019–1024. 10.4315/0362-028x-64.7.1019 11456186

[B82] Emami N. K. Samie A. Rahmani H. R. Ruiz-Feria C. A. (2012). The effect of peppermint essential oil and fructooligosaccharides, as alternatives to virginiamycin, on growth performance, digestibility, gut morphology and immune response of male broilers. *Anim. Feed Sci. Technol.* 175 57–64. 10.1016/j.anifeedsci.2012.04.001

[B83] Epps S. V. Harvey R. B. Byrd J. A. Petrujkiæ B. T. Sedej I. Beier R. C. (2015). Comparative effect of thymol or its glucose conjugate, thymol-β-D-glucopyranoside, on *Campylobacter* in avian gut contents. *J. Environ. Sci. Health Part B* 50 55–61. 10.1080/03601234.2015.965634 25421628

[B84] European Food Safety Authority [EFSA] (2010). Scientific opinion on quantification of the risk posed by broiler meat to human campylobacteriosis in the EU. *EFSA J.* 8:1437 10.2903/j.efsa.2010.1437 PMC13129989 42077633

[B85] Evans W. C. Fuchs G. (1988). Anaerobic degradation of aromatic compounds. *Annu. Rev. Microbiol.* 42 289–317. 10.1146/annurev.micro.42.1.289 3059996

[B86] Fabricant F. (2008). *Earl Grey Flavor, to Serve after Teatime.* New York, NY: New York Times, D2.

[B87] Facciolà A. Riso R. Avventuroso E. Visalli G. Delia S. A. Laganà P. (2017). *Campylobacter*: from microbiology to prevention. *J. Prev. Med. Hyg.* 58:E79. PMC5584092 28900347

[B88] Fenton W. A. Horwich A. L. (1997). GroEL mediated protein folding. *Prot. Sci.* 6 743–760. 10.1002/pro.5560060401 9098884 PMC2144759

[B89] Fisher K. Phillips C. (2008). Potential antimicrobial uses of essential oils in food: is citrus the answer? *Trends Food Sci. Technol.* 19 156–164. 10.1016/j.tifs.2007.11.006

[B90] Fisher K. Phillips C. (2009). The mechanism of action of a citrus oil blend against *Enterococcus faecium* and *Enterococcus faecalis*. *J. Appl. Microbiol.* 106 1343–1349. 10.1111/j.1365-2672.2008.04102.x 19187138

[B91] Fisher K. Phillips C. A. (2006). The effect of lemon, orange and bergamot essential oils and their components on the survival of *Campylobacter jejuni, Escherichia coli* O157, *Listeria monocytogenes, Bacillus cereus* and *Staphylococcus aureus in vitro* and in food systems. *J. Appl. Microbiol.* 101 1232–1240. 10.1111/j.1365-2672.2006.03035.x 17105553

[B92] Fitzgerald C. Nachamkin I. (2011). “*Campylobacter* and *Arcobacter*,” in *Manual of Clinical Microbiology*, eds Versalovic J. Carroll K. Funke G. Jorgensen J. Landry M. L. Warnock D. W. (Washington, DC: ASM Press), 885–899.

[B93] Frank J. F. Ehlers J. Wicker L. (2003). Removal of *Listeria monocytogenes* and poultry soil-containing biofilms using chemical cleaning and sanitizing agents under static conditions. *Food Prot. Trends* 23 654–663.

[B94] Franz C. Baser K. H. C. Windisch W. (2010). Essential oils and aromatic plants in animal feeding–a European perspective. A review. *Flavour Fragr. J.* 25 327–340. 10.1002/ffj.1967

[B95] Frazer A. C. (1994). “O-demethylation and other transformations of aromatic compounds by acetogenic bacteria,” in *Acetogenesis*, ed. Drake H. L. (Boston, MA: Springer), 445–483. 10.1007/978-1-4615-1777-1_17

[B96] Friedman C. R. Hoekstra R. M. Samuel M. Marcus R. Bender J. Shiferaw B. (2004). Risk factors for sporadic *Campylobacter* infection in the United States: a case-control study in FoodNet sites. *Clin. Infect. Dis.* 38(Suppl._3), S285–S296. 15095201 10.1086/381598

[B97] Friedman M. Henika P. R. Mandrell R. E. (2002). Bactericidal activities of plant essential oils and some of their isolated constituents against *Campylobacter jejuni, Escherichia coli, Listeria monocytogenes*, and *Salmonella enterica*. *J. Food Prot.* 65 1545–1560. 10.4315/0362-028x-65.10.1545 12380738

[B98] Gangaiah D. Liu Z. Arcos J. Kassem I. I. Sanad Y. Torrelles J. B. (2010). Polyphosphate kinase 2: a novel determinant of stress responses and pathogenesis in *Campylobacter jejuni*. *PLoS One* 5:e12142. 10.1371/journal.pone.0012142 20808906 PMC2923150

[B99] Garénaux A. Jugiau F. Rama F. De Jonge R. Denis M. Federighi M. (2008). Survival of *Campylobacter jejuni* strains from different origins under oxidative stress conditions: effect of temperature. *Curr. Microbiol.* 56 293–297. 10.1007/s00284-007-9082-8 18180992

[B100] Geissler C. Powers H. (2011). “Chapter 3: food safety,” in *Human Nutrition*, 12th Edn, eds Geissler C. Powers H. (Philadelphia, PA: Churchill Livingstone Elsevier), 51–68.

[B101] Gerwe T. Bouma A. Wagenaar J. A. Jacobs-Reitsma W. F. Stegeman A. (2010). Comparison of *Campylobacter* levels in crops and ceca of broilers at slaughter. *Avian Dis.* 54 1072–1074. 10.1637/9113-101809-resnote.1 20945790

[B102] Gongqiao X. U. Khatri I. A. Wang R. Rahman S. Forstner J. F. (2003). N-linked oligosaccharides play a role in disulphide-dependent dimerization of intestinal mucin Muc2. *Biochem. J.* 373 893–900. 10.1042/bj20030096 12744721 PMC1223556

[B103] Gonzalez-Molina E. Moreno D. A. Garcia-Viguera C. (2009). A new drink rich in healthy bioactives combining lemon and pomegranate juices. *Food Chem.* 115 1364–1372. 10.1016/j.foodchem.2009.01.056

[B104] Gous R. M. (2010). Nutritional limitations on growth and development in poultry. *Livest. Sci.* 130 25–32. 10.1016/j.livsci.2010.02.007

[B105] Gracia M. I. Millan C. Sanchez J. Guyard-Nicodeme M. Mayot J. Carre Y. (2015). Efficacy of feed additives against *Campylobacter* in live broilers during the entire rearing period: part B. *Poult. Sci.* 95 886–892. 10.3382/ps/pev346 26706354

[B106] Grand View Research (2018). *Essential Oils Market Size, Share & Trends Analysis Report By Product (Orange, Corn, Mint, Eucalyptus, Citronella, Pepper Mint, Lemon, Clove Leaf, Lime, Spearmint), By Application, And Segment Forecasts, 2018 - 2025.*

[B107] Grilli E. Vitari F. Domeneghini C. Palmonari A. Tosi G. Fantinati P. (2013). Development of a feed additive to reduce caecal *Campylobacter jejuni* in broilers at slaughter age: from *in vitro* to *in vivo*, a proof of concept. *J. Appl. Microbiol.* 114 308–317. 10.1111/jam.12053 23110383

[B108] Guarner F. Malagelada J. R. (2003). Gut flora in health and disease. *Lancet* 361 512–519. 10.1016/s0140-6736(03)12489-0 12583961

[B109] Guccione E. Del Rocio Leon-Kempis M. Pearson B. M. Hitchin E. Mulholland F. van Diemen P. M. (2008). Amino acid-dependent growth of *Campylobacter jejuni:* key roles for aspartase (AspA) under microaerobic and oxygen-limited conditions and identification of AspB (Cj0762), essential for growth on glutamate. *Mol. Microbiol.* 69 77–93. 10.1111/j.1365-2958.2008.06263.x 18433445

[B110] Guenther E. (ed.) (1948). “The essential oils,” in *Essential Oils*, (New York, NY: D. Van Nostrand), 8–52.

[B111] Guinoiseau E. Luciani A. Rossi P. G. Quilichini Y. Ternengo S. Bradesi P. (2010). Cellular effects induced by *Inula graveolens* and *Santolina corsica* essential oils on *Staphylococcus aureus*. *Eur. J. Clin. Microbiol. Infect. Dis.* 29 873–879. 10.1007/s10096-010-0943-x 20490884

[B112] Gunther N. W. Chen C. Y. (2009). The biofilm forming potential of bacterial species in the genus *Campylobacter*. *Food Microbiol.* 26 44–51. 10.1016/j.fm.2008.07.012 19028304

[B113] Guyard-Nicodeme M. Keita A. Quesne S. Amelot M. Poezevara T. Le Berre B. (2015). Efficacy of feed additives against *Campylobacter* in live broilers during the entire rearing period. *Poult. Sci.* 95 298–305. 10.3382/ps/pev303 26706356

[B114] Häggblom M. M. Rivera M. D. Young L. Y. (1993). Influence of alternative electron acceptors on the anaerobic biodegradability of chlorinated phenols and benzoic acids. *Appl. Environ. Microbiol.* 59 1162–1167. 8476290 10.1128/aem.59.4.1162-1167.1993 PMC202255

[B115] Hajlaoui H. Trabelsi N. Noumi E. Snoussi M. Fallah H. Ksouri R. (2009). Biological activities of the essential oils and methanol extract of two cultivated mint species (*Mentha longifolia* and *Mentha pulegium*) used in the Tunisian folkloric medicine. *World J. Microbiol. Biotechnol.* 25 2227–2238. 10.1007/s11274-009-0130-3

[B116] Hansen A. K. Hansen C. H. F. Krych L. Nielsen D. S. (2014). Impact of the gut microbiota on rodent models of human disease. *World J. Gastroenterol.* 20:17727. 10.3748/wjg.v20.i47.17727 25548471 PMC4273123

[B117] Hardin A. Crandall P. G. Stankus T. (2010). Essential oils and antioxidants derived from citrus by-products in food protection and medicine: an introduction and review of recent literature. *J. Agric. Food Inf.* 11 99–122. 10.1080/10496501003680680

[B118] Hardy H. Harris J. Lyon E. Beal J. Foey A. (2013). Probiotics, prebiotics and immunomodulation of gut mucosal defences: homeostasis and immunopathology. *Nutrients* 5 1869–1912. 10.3390/nu5061869 23760057 PMC3725482

[B119] Hargis B. M. Caldwell D. J. Brewer R. L. Corrier D. E. DeLoach J. R. (1995). Evaluation of the chicken crop as a source of *Salmonella* contamination for broiler carcasses. *Poult. Sci.* 74 1548–1552. 10.3382/ps.0741548 7501601

[B120] Hazeleger W. Wouters J. Rombuts F. Abee T. (1998). Physiological activity of *Campylobacter jejuni* far below the minimal growth temperature. *Appl. Environ. Microbiol.* 64 3917–3922. 9758819 10.1128/aem.64.10.3917-3922.1998 PMC106578

[B121] Helander I. M. Alakomi H. L. Latva-Kala K. Mattila-Sandholm T. Pol I. Smid E. J. (1998). Characterization of the action of selected essential oil components on Gram-negative bacteria. *J. Agric. Food Chem.* 46 3590–3595. 10.1021/jf980154m

[B122] Hermans D. Martel A. Van Deun K. Verlinden M. Van Immerseel F. Garmyn A. (2010). Intestinal mucus protects *Campylobacter jejuni* in the ceca of colonized broiler chickens against the bactericidal effects of medium-chain fatty acids. *Poult. Sci.* 89 1144–1155. 10.3382/ps.2010-00717 20460660

[B123] Hermans D. Pasmans F. Heyndrickx M. Van Immerseel F. Martel A. Van Deun K. (2012). A tolerogenic mucosal immune response leads to persistent *Campylobacter jejuni* colonization in the chicken gut. *Crit. Rev. Microbiol.* 38 17–29. 10.3109/1040841X.2011.615298 21995731

[B124] Hermans D. Martel A. Van Deun K. Van Immerseel F. Heyndrickx M. Haesebrouck F. (2011a). The cinnamon-oil ingredient trans-cinnamaldehyde fails to target *Campylobacter jejuni* strain KC 40 in the broiler chicken cecum despite marked in vitro activity. *J. Food Prot.* 74 1729–1734. 10.4315/0362-028X.JFP-10-487 22004822

[B125] Hermans D. Van Deun K. Martel A. Van Immerseel F. Messens W. Heyndrickx M. (2011b). Colonization factors of *Campylobacter jejuni* in the chicken gut. *Vet. Res.* 42:82. 10.1186/1297-9716-42-82 21714866 PMC3156733

[B126] Hernandez F. Madrid J. Garcia V. Orengo J. Megias M. D. (2004). Influence of two plant extracts on broilers performance, digestibility, and digestive organ size. *Poult. Sci.* 83 169–174. 10.1093/ps/83.2.169 14979566

[B127] Hinton A. (2006). Growth of *Campylobacter* in media supplemented with organic acids. *J. Food Prot.* 69 34–38. 10.4315/0362-028x-69.1.34 16416898

[B128] Hofreuter D. Tsai J. Watson R. O. Novik V. Altman B. Benitez M. (2006). Unique features of a highly pathogenic *Campylobacter jejuni* strain. *Infect. Immun.* 74 4694–4707. 10.1128/iai.00210-06 16861657 PMC1539605

[B129] Holley R. A. Patel D. (2005). Improvement in shelf-life and safety of perishable foods by plant essential oils and smoke antimicrobials. *Food Microbiol.* 22 273–292. 10.1016/j.fm.2004.08.006

[B130] Hooper L. V. Wong M. H. Thelin A. Hansson L. Falk P. G. Gordon J. I. (2001). Molecular analysis of commensal host-microbial relationships in the intestine. *Science* 291 881–884. 10.1126/science.291.5505.881 11157169

[B131] Horrocks S. M. Anderson R. C. Nielsbet D. J. Ricke S. C. (2009). Incidence and ecology of *Campylobacter jejuni* and coli in animals. *Anaerobe* 15 18–25. 10.1016/j.anaerobe.2008.09.001 18849005

[B132] Hue O. Le Bouquin S. Laisney M. J. Allain V. Lalande F. Petetin I. (2010). Prevalence of and risk factors for *Campylobacter* spp. contamination of broiler chicken carcasses at the slaughterhouse. *Food Microbiol.* 27 992–999. 10.1016/j.fm.2010.06.004 20832676

[B133] Hugdahl M. B. Beery J. T. Doyle M. P. (1988). Chemotactic behavior of *Campylobacter jejuni*. *Infect. Immun.* 56 1560–1566. 3372020 10.1128/iai.56.6.1560-1566.1988 PMC259436

[B134] Hyldgaard M. Mygind T. Meyer R. L. (2012). Essential oils in food preservation: mode of action, synergies, and interactions with food matrix components. *Front. Microbiol.* 3:12. 10.3389/fmicb.2012.00012 22291693 PMC3265747

[B135] Indikova I. Humphrey T. J. Hilbert F. (2015). Survival with a helping hand: *Campylobacter* and microbiota. *Front. Microbiol.* 6:1266. 10.3389/fmicb.2015.01266 26617600 PMC4637420

[B136] Jager W. (2010). “Metabolism of terpenoids in animal models and humans,” in *Handbook of Essential Oils: Science, Technology, and Applications*, eds Baser K. H. C. Buchbauer G. (Boca Raton, FL: CRC Press), 209–235.

[B137] Jahrmann R. (2007). *Metabolismus von Monterpenen und Sesquiterpenen in Mensch und Säugetier: Bedeutung für die Pharmazeutische Praxis. MPharm.* Diploma thesis, University of Vienna, Vienna.

[B138] Jamroz D. Orda J. Kamel C. Wiliczkiewicz A. Wertelecki T. Skorupinska J. (2003). The influence of phytogenic extracts on performance, nutrient digestibility, carcass characteristics, and gut microbial status in broiler chickens. *J. Anim. Feed Sci.* 12 583–596. 10.22358/jafs/67752/2003

[B139] Jamroz D. Wertelecki T. Houszka M. Kamel C. (2006). Influence of diet type on the inclusion of plant origin active substances on morphological and histochemical characteristics of the stomach and jejunum walls in chicken. *J. Anim. Physiol. Anim. Nutr.* 90 255–268. 10.1111/j.1439-0396.2005.00603.x 16684147

[B140] Jang I. S. Ko Y. H. Kang S. Y. Lee C. Y. (2007). Effect of a commercial essential oil on growth performance, digestive enzyme activity and intestinal microflora population in broiler chickens. *Anim. Feed Sci. Technol.* 134 304–315. 10.1016/j.anifeedsci.2006.06.009

[B141] Jang I. S. Ko Y. H. Yang H. Y. Ha J. S. Kim J. Y. Kang S. Y. (2004). Influence of essential oil components on growth performance and the functional activity of the pancreas and small intestine in broiler chickens. *Asian Australas. J. Anim. Sci.* 17 394–400. 10.5713/ajas.2004.394

[B142] Jo C. Kang H. J. Lee M. Lee N. Y. Byun M. W. (2004). The antioxidative potential of lyophilized citrus peel extract in different meat model systems during storage at 20 C. *J. Muscle Foods* 15 95–107. 10.1111/j.1745-4573.2004.tb00714.x

[B143] Johnson L. P. Walton G. E. Psichas A. Frost G. S. Gibson G. R. Barraclough T. G. (2015). Prebiotics modulate the effects of antibiotics on gut microbial diversity and functioning in vitro. *Nutrients* 7 4480–4497. 10.3390/nu7064480 26053617 PMC4488797

[B144] Johnson R. (2015). *US-EU Poultry Dispute on the use of Pathogen Reduction Treatments (PRTs).* Washington, DC: Congressional Research Service, 7–57.

[B145] Józefiak D. Rutkowski A. Martin S. A. (2004). Carbohydrate fermentation in the avian ceca: a review. *Anim. Feed Sci. Technol.* 113 1–15. 10.1016/j.anifeedsci.2003.09.007 29955220

[B146] Juliano C. Mattana A. Usai M. (2000). Composition and in vitro antimicrobial activity of the essential oil of Thymus herba-barona Loisel growing wild in Sardinia. *J. Essent. Oil Res.* 12 516–522. 10.1080/10412905.2000.9699578

[B147] Jung H. G. Fahey G. C. Jr. (1983). Nutritional implications of phenolic monomers and lignin: a review 1. *J. Anim. Sci.* 57 206–219. 10.2527/jas1983.571206x

[B148] Jyothi S. S. Seethadevi A. Prabha K. S. Muthuprasanna P. Pavitra P. (2012). Microencapsulation: a review. *Int. J. Pharma Bio Sci.* 3 509–531.

[B149] Kaakoush N. O. Sodhi N. Chenu J. W. Cox J. M. Riordan S. M. Mitchell H. M. (2014). The interplay between *Campylobacter* and *Helicobacter* species and other gastrointestinal microbiota of commercial broiler chickens. *Gut Pathog.* 6:18. 10.1186/1757-4749-6-18 24940386 PMC4060860

[B150] Kalchayanand N. Dunne P. Sikes A. Ray B. (2004). Viability loss and morphology change of foodborne pathogens following exposure to hydrostatic pressures in the presence and absence of bacteriocins. *Int. J. Food Microbiol.* 91 91–98. 10.1016/s0168-1605(03)00324-6 14967564

[B151] Kassem I. I. Chandrashekhar K. Rajashekara G. (2013). Of energy and survival incognito: a relationship between viable but non-culturable cells formation and inorganic polyphosphate and formate metabolism in *Campylobacter jejuni*. *Front. Microbiol.* 4:183. 10.3389/fmicb.2013.00183 23847606 PMC3705167

[B152] Kavanaugh N. L. Ribbeck K. (2012). Selected antimicrobial essential oils eradicate *Pseudomonas* spp. and *Staphylococcus aureus* biofilms. *Appl. Environ. Microbiol.* 78 4057–4061. 10.1128/AEM.07499-11 22467497 PMC3346404

[B153] Keener K. M. Bashor M. P. Curtis P. A. Sheldon B. W. Kathariou S. (2004). Comprehensive review of *Campylobacter* and poultry processing. *Compr. Rev. Food Sci. Food Saf.* 3 105–116. 10.1111/j.1541-4337.2004.tb00060.x 33430546

[B154] Kelly C. Gundogdu O. Pircalabioru G. Cean A. Scates P. Linton M. (2017). The *in vitro* and *in vivo* effect of carvacrol in preventing *Campylobacter* infection, colonization and in improving productivity of chicken broilers. *Foodborne Pathog. Dis.* 14 341–349. 10.1089/fpd.2016.2265 28398869

[B155] Kerekes E. B. Vidács A. Török Jenei J. Gömöri C. Takó M. Chandrasekaran M. (2015). “Essential oils against bacterial biofilm formation and quorum sensing of food-borne pathogens and spoilage microorganisms,” in *The Battle Against Microbial Pathogens: Basic Science, Technological Advances and Educational Programs. Microbiology Book Series* Vol. 5 ed. Méndez-Vilas A. (Bajadoz: Formatex Research Center), 429–437.

[B156] Keto-Timonen R. Hietala N. Palonen E. Hakakorpi A. Lindström M. Korkeala H. (2016). Cold shock proteins: a minireview with special emphasis on Csp-family of enteropathogenic *Yersinia*. *Front. Microbiol.* 7:1151. 10.3389/fmicb.2016.01151 27499753 PMC4956666

[B157] Khan I. A. Abourashed E. A. (2011). *Leung’s Encyclopedia of Common Natural Ingredients: Used in Food, Drugs and Cosmetics.* Hoboken, NJ: John Wiley & Sons.

[B158] Kirkpinar F. Ünlü H. B. Serdaroğlu M. Turp G. Y. (2014). Effects of dietary oregano and garlic essential oils on carcass characteristics, meat composition, colour, pH and sensory quality of broiler meat. *Br. Poult. Sci.* 55 157–166. 10.1080/00071668.2013.879980 24404997

[B159] Kivanç M. Akgül A. Dogan A. (1991). Inhibitory and stimulatory effects of cumin, oregano and their essential oils on growth and acid production of *Lactobacillus plantarum* and *Leuconostoc mesenteroides*. *Int. J. Food Microbiol.* 13 81–85. 10.1016/0168-1605(91)90140-k 1863531

[B160] Klancnik A. Guzej B. Kolar H. M. Abramovic H. Smole Mozina S. (2009). In vitro antimicrobial and antioxidant activity of commercial rosemary extract formulations. *J. Food Prot.* 72 1744–1752. 10.4315/0362-028x-72.8.1744 19722413

[B161] Kohlert C. Schindler G. Marz R. W. Abel G. Brinkhaus B. Derendorf H. (2002). Systemic availability and pharmacokinetics of thymol in humans. *J. Clin. Pharmocol.* 42 731–737. 10.1177/009127002401102678 12092740

[B162] Kollanoor-Johny A. Darre M. J. Donoghue A. M. Donoghue D. J. Venkitanarayanan K. (2010). Antibacterial effect of trans-cinnamaldehyde, eugenol, carvacrol, and thymol on *Salmonella* Enteritidis and *Campylobacter jejuni* in chicken cecal contents in vitro. *J. Appl. Poult. Res.* 19 237–244. 10.3382/japr.2010-00181

[B163] Konkel M. E. Christensen J. E. Keech A. M. Monteville M. R. Klena J. D. Garvis S. G. (2005). Identification of a fibronectin-binding domain within the *Campylobacter jejuni* CadF protein. *Mol. Microbiol.* 57 1022–1035. 10.1111/j.1365-2958.2005.04744.x 16091041

[B164] Kostaki M. Giatrakou V. Savvaidis I. N. Kontominas M. G. (2009). Combined effect of MAP and thyme essential oil on the microbiological, chemical and sensory attributes of organically aquacultured sea bass (*Dicentrarchus labrax*) fillets. *Food Microbiol.* 26 475–482. 10.1016/j.fm.2009.02.008 19465243

[B165] Koutsos E. A. Arias V. J. (2006). Intestinal ecology: interactions among the gastrointestinal tract, nutrition, and the microflora. *J. Appl. Poult. Res.* 15 161–173. 10.1093/japr/15.1.161

[B166] Kreydiyyeh S. I. Usta J. Copti R. (2000). Effect of cinnamon, clove and some of their constituents on the Na+-K+-ATPase activity and alanine absorption in the rat jejunum. *Food Chem. Toxicol.* 38 755–762. 10.1016/s0278-6915(00)00073-9 10930696

[B167] Kroismayr A. Sehm J. Pfaffl M. W. Schedle K. Plitzner C. Windisch W. (2008). Effects of avilamycin and essential oils on mRNA expression of apoptotic and inflammatory markers and gut morphology of piglets. *Czech J. Anim. Sci.* 53 377–387. 10.17221/338-cjas

[B168] Kumar J. K. Tabor S. Richardson C. C. (2004). Proteomic analysis of thioredoxin-targeted proteins in *Escherichia coli*. *Proc. Natl. Acad. Sci. U.S.A.* 101 3759–3764. 10.1073/pnas.0308701101 15004283 PMC374317

[B169] Kurekci C. Padmanabha J. Bishop-Hurley S. L. Hassan E. Al Jassim R. A. McSweeney C. S. (2013). Antimicrobial activity of essential oils and five terpenoid compounds against *Campylobacter jejuni* in pure and mixed culture experiments. *Int. J. Food Microbiol.* 166 450–457. 10.1016/j.ijfoodmicro.2013.08.014 24041998

[B170] Kwon J. A. Yu C. B. Park H. D. (2003). Bacteriocidal effects and inhibition of cell separation of cinnamic aldehyde on *Bacillus cereus*. *Lett. Appl. Microbiol.* 37 61–65. 10.1046/j.1472-765x.2003.01350.x 12803558

[B171] Laanbroek H. J. Kingma W. Veldkamp H. (1977). Isolation of an aspartate fermenting, free-living *Campylobacter* species. *FEMS Microbiol. Lett.* 1 99–102. 10.1016/0378-1097(77)90010-6

[B172] Lambert R. J. W. Skandamis P. N. Coote P. J. Nychas G. J. (2001). A study of the minimum inhibitory concentration and mode of action of oregano essential oil, thymol and carvacrol. *J. Appl. Microbiol.* 91 453–462. 10.1046/j.1365-2672.2001.01428.x 11556910

[B173] Lee H. W. Park Y. S. Jung J. S. Shin W. S. (2002). Chitosan oligosaccharides, dp 2–8, have prebiotic effect on the *Bifidobacterium bifidum* and *Lactobacillus* sp. *Anaerobe* 8 319–324. 10.1016/s1075-9964(03)00030-1 16887676

[B174] Lee K. W. Everts H. Beynen A. (2004a). Essential oils in broiler nutrition. *Int. J. Poult. Sci.* 3 738–752. 10.3923/ijps.2004.738.752

[B175] Lee K. W. Everts H. Kappert H. J. van Der Kuilen J. Lemmens A. G. Frehner M. (2004b). Growth performance, intestinal viscosity, fat digestibility and plasma cholesterol in broiler chickens fed a rye-containing diet without or with essential oil components. *Int. J. Poult. Sci.* 3 613–618. 10.3923/ijps.2004.613.618

[B176] Lee K. W. Everts H. Kappert H. J. Frehner M. Losa R. Beynen A. C. (2003). Effects of dietary essential oil components on growth performance, digestive enzymes and lipid metabolism in female broiler chickens. *Br. Poult. Sci.* 44 450–457. 10.1080/00071660301985 12964629

[B177] Leenstra F. R. (1986). Effect of age, sex, genotype and environment on fat deposition in broiler chickens—a review. *Worlds Poult. Sci. J.* 42 12–25. 10.1079/wps19860002 1956848

[B178] Leistner L. (2000). Basic aspects of food preservation by hurdle technology. *Int. J. Food Microbiol.* 55 181–186. 10.1016/s0168-1605(00)00161-6 10791741

[B179] Levin R. E. (2007). *Campylobacter jejuni*: a review of its characteristics, pathogenicity, ecology, distribution, subspecies characterization and molecular methods of detection. *Food Biotechnol.* 21 271–347. 10.1080/08905430701536565

[B180] Lien K. A. Sauer W. C. He J. M. (2001). Dietary influences on the secretion into and degradation of mucin in the digestive tract of monogastric animals and humans. *J. Anim. Feed Sci.* 10 223–246.

[B181] Lin A. E. Krastel K. Hobb R. I. Thompson S. A. Cvitkovitch D. G. Gaynor E. C. (2009). Atypical roles for *Campylobacter jejuni* amino acid ATP binding cassette transporter components PaqP and PaqQ in bacterial stress tolerance and pathogen-host cell dynamics. *Infect. Immunol.* 77 4912–4924. 10.1128/IAI.00571-08 19703978 PMC2772506

[B182] Line J. E. (2001). Development of a selective differential agar for isolation and enumeration of *Campylobacter* spp. *J. Food Prot.* 64 1711–1715. 10.4315/0362-028x-64.11.1711 11726148

[B183] Line J. E. Hiett K. L. Guard-Bouldin J. Seal B. S. (2010). Differential carbon source utilization by *Campylobacter jejuni* 11168 in response to growth temperature variation. *J. Microbiol. Methods* 80 198–202. 10.1016/j.mimet.2009.12.011 20035808

[B184] Liu S. Bennett D. C. Tun H. M. Kim J. E. Cheng K. M. Zhang H. (2015). The effect of diet and host genotype on ceca microbiota of Japanese quail fed a cholesterol enriched diet. *Front. Microbiol.* 6:1092. 10.3389/fmicb.2015.01092 26500632 PMC4595795

[B185] Looft T. Cai G. Choudhury B. Lai L. X. Lippolis J. D. Reinhardt T. A. (2019). Avian intestinal mucus modulates *Campylobacter jejuni* gene expression in a host-specific manner. *Front. Microbiol.* 9:3215. 10.3389/fmicb.2018.03215 30687245 PMC6338021

[B186] Los Santos F. S. Donoghue A. M. Venkitanarayanan K. Dirain M. L. Reyes-Herrera I. Blore P. J. (2008). Caprylic acid supplemented in feed reduces enteric *Campylobacter jejuni* colonization in ten-day-old broiler chickens. *Poult. Sci.* 87 800–804. 10.3382/ps.2007-00280 18340004

[B187] Los Santos F. S. Donoghue A. M. Venkitanarayanan K. Metcalf J. H. Reyes-Herrera I. Dirain M. L. (2009). The natural feed additive caprylic acid decreases *Campylobacter jejuni* colonization in market-aged broiler chickens. *Poult. Sci.* 88 61–64. 10.3382/ps.2008-00228 19096058

[B188] Lourenço M. C. Kuritza L. N. Hayashi R. M. Miglino L. B. Durau J. F. Pickler L. (2015). Effect of a mannanoligosaccharide-supplemented diet on intestinal mucosa T lymphocyte populations in chickens challenged with *Salmonella* Enteritidis. *J. Appl. Poult. Res.* 24 15–22. 10.3382/japr/pfu002

[B189] Mack D. R. Michail S. Wei S. McDougall L. Hollingsworth M. A. (1999). Probiotics inhibit enteropathogenic *E. coli* adherence in vitro by inducing intestinal mucin gene expression. *Am. J. Physiol.* 276 G941–G950. 10.1152/ajpgi.1999.276.4.G941 10198338

[B190] Manzanilla E. G. Baucells F. Kamel C. Morales J. Perez J. F. Gasa J. (2001). Effects of plant extracts on the performance and lower gut microflora of early weaned piglets. *J. Anim. Sci.* 1:473. 10.1111/jpn.12976 30129225

[B191] Manzanilla E. G. Perez J. F. Martin M. Kamel C. Baucells F. Gasa J. (2004). Effect of plant extracts and formic acid on the intestinal equilibrium of early– weaned pigs. *J. Anim. Sci.* 82 3210–3218. 10.2527/2004.82113210x 15542467

[B192] Martucci J. F. Gende L. B. Neira L. M. Ruseckaite R. A. (2015). Oregano and lavender essential oils as antioxidant and antimicrobial additives of biogenic gelatin films. *Ind. Crops Prod.* 71 205–213. 10.1016/j.indcrop.2015.03.079

[B193] Messaoudi S. Kergourlay G. Dalgalarrondo M. Choiset Y. Ferchichi M. Prévost H. (2012). Purification and characterization of a new bacteriocin active against *Campylobacter* produced by *Lactobacillus salivarius* SMXD51. *Food Microbiol.* 32 129–134. 10.1016/j.fm.2012.05.002 22850384

[B194] Metcalf J. H. Donoghue A. M. Venkitanarayanan K. Reyes-Herrera I. Aguiar V. F. Blore P. J. (2011). Water administration of the medium-chain fatty acid caprylic acid produced variable efficacy against enteric *Campylobacter* colonization in broilers. *Poult. Sci.* 90 494–497. 10.3382/ps.2010-00891 21248350

[B195] Meunier J. P. Cardot J. M. Gauthier P. Beyssac E. Alric M. (2006). Use of rotary fluidized-bed technology for development of sustained-release plant extracts pellets: potential application for feed additive delivery. *J. Anim. Sci.* 84 1850–1859. 10.2527/jas.2005-361 16775069

[B196] Micciche A. C. Foley S. L. Pavlidis H. O. McIntyre D. R. Ricke S. C. (2018a). A review of prebiotics against *Salmonella* in poultry: current and future potential for microbiome research applications. *Front. Vet. Sci.* 5:191. 10.3389/fvets.2018.00191 30159318 PMC6104193

[B197] Micciche A. C. Rubinelli P. M. Ricke S. C. (2018b). Source of water and potential sanitizers and biological antimicrobials for alternative poultry processing food safety applications. *Front. Sustain. Food Syst.* 2:82 10.3389/fsufs.2018.00082

[B198] Michiels J. Missotten J. Dierick N. Fremaut D. Maene P. De Smet S. (2008). *In vitro* degradation and *in vivo* passage kinetics of carvacrol, thymol, eugenol and trans-cinnamaldehyde along the gastrointestinal tract of piglets. *J. Sci. Food Agric.* 88 2371–2381. 10.1002/jsfa.3358

[B199] Mohammadhosseini M. Sarker S. D. Akbarzadeh A. (2017). Chemical composition of the essential oils and extracts of *Achillea* species and their biological activities: a review. *J. Ethnopharmacol.* 199 257–315. 10.1016/j.jep.2017.02.010 28179115

[B200] Moleyar V. Narasimham P. (1992). Antibacterial activity of essential oil components. *Int. J. Food Microbiol.* 16 337–342. 10.1016/0168-1605(92)90035-2 1457292

[B201] Montagne L. Pluske J. R. Hampson D. J. (2003). A review of interactions between dietary fibre and the intestinal mucosa, and their consequences on digestive health in young non-ruminant animals. *Anim. Feed Sci. Technol.* 108 95–117. 10.1016/s0377-8401(03)00163-9

[B202] Monteville M. R. Yoon J. E. Konkel M. E. (2003). Maximal adherence and invasion of INT 407 cells by *Campylobacter jejuni* requires the CadF outer-membrane protein and microfilament reorganization. *Microbiology* 149 153–165. 10.1099/mic.0.25820-0 12576589

[B203] Montrose M. S. Shane S. M. Harrington K. S. (1985). Role of litter in the transmission of *Campylobacter jejuni*. *Avian Dis.* 29 392–399. 4026733

[B204] Moreira M. R. Ponce A. G. Del Valle C. E. Roura S. I. (2005). Inhibitory parameters of essential oils to reduce a foodborne pathogen. *LWT Food Sci. Technol.* 38 565–570. 10.1016/j.lwt.2004.07.012

[B205] Motojima F. (2015). How do chaperonins fold protein? *Biophysics* 11 93–102. 10.2142/biophysics.11.93 27493521 PMC4736790

[B206] Muraoka W. T. Zhang Q. (2010). Phenotypic and genotypic evidence for L-fucose utilization by *Campylobacter jejuni*. *J. Bacteriol.* 193 1065–1075. 10.1128/JB.01252-10 21193610 PMC3067607

[B207] Murray C. J. Barber R. M. Foreman K. J. Ozgoren A. A. Abd-Allah F. Abera S. F. (2015). Global, regional, and national disability-adjusted life years (DALYs) for 306 diseases and injuries and healthy life expectancy (HALE) for 188 countries, 1990–2013: quantifying the epidemiological transition. *Lancet* 386 2145–2191. 26321261 10.1016/S0140-6736(15)61340-X PMC4673910

[B208] Nakatsu T. Lupo A. T. Jr. Chinn J. W. Jr. Kang R. K. (2000). “Biological activity of essential oils and their constituents,” in *Studies in Natural Products Chemistry* Vol. 21 ed. Rahman A. (Amsterdam: Elsevier), 571–631. 10.1016/s1572-5995(00)80014-9

[B209] Namkung H. Li J. Gong M. Yu H. Cottrill M. De Lange C. F. M. (2004). Impact of feeding blends of organic acids and herbal extracts on growth performance, gut microbiota and digestive function in newly weaned pigs. *Can. J. Anim. Sci.* 84 697–704. 10.4141/a04-005

[B210] Nannapaneni R. Chalova V. I. Crandall P. G. Ricke S. C. Johnson M. G. O’Bryan C. A. (2009). *Campylobacter* and *Arcobacter* species sensitivity to commercial orange oil fractions. *Int. J. Food Microbiol.* 129 43–49. 10.1016/j.ijfoodmicro.2008.11.008 19070381

[B211] National Organic Program (2018). National Organic Program, 7 C.F.R. 205.601-603.

[B212] Naughton J. A. Mariño K. Dolan B. Reid C. Gough R. Gallagher M. E. (2013). Divergent mechanisms of interaction of *Helicobacter pylori* and *Campylobacter jejuni* with mucus and mucins. *Infect. Immun.* 81 2838–2850. 10.1128/IAI.00415-13 23716616 PMC3719574

[B213] Nauta M. Hill A. Rosenquist H. Brynestad S. Fetsch A. van der Logt P. (2009). A comparison of risk assessments on *Campylobacter* in broiler meat. *Int. J. Food Microbiol.* 129 107–123. 10.1016/j.ijfoodmicro.2008.12.001 19136176

[B214] Navarro M. Stanley R. Cusack A. Sultanbawa Y. (2015). Combinations of plant-derived compounds against *Campylobacter* in vitro. *J. Appl. Poult. Res.* 24 352–363. 10.3382/japr/pfv035

[B215] Nazzaro F. Fratianni F. De Martino L. Coppola R. De Feo V. (2013). Effect of essential oils on pathogenic bacteria. *Pharmaceuticals* 6 1451–1474. 10.3390/ph6121451 24287491 PMC3873673

[B216] Netzer F. Kuntze K. Vogt C. Richnow H. H. Boll M. Lueders T. (2016). Functional gene markers for fumarate-adding and dearomatizing key enzymes in anaerobic aromatic hydrocarbon degradation in terrestrial environments. *J. Mol. Microbiol. Biotechnol.* 26 180–194. 10.1159/000441946 26959523

[B217] Nguefack J. Tamgue O. Dongmo J. B. L. Dakole C. D. Leth V. Vismer H. F. (2012). Synergistic action between fractions of essential oils from *Cymbopogon citratus, Ocimum gratissimum* and *Thymus vulgaris* against *Penicillium expansum*. *Food Control* 23 377–383. 10.1016/j.foodcont.2011.08.002

[B218] Nofrarias M. Manzanilla E. G. Pujols J. Gibert X. Majo N. Segalés J. (2006). Effects of spray-dried porcine plasma and plant extracts on intestinal morphology and on leukocyte cell subsets of weaned pigs. *J. Anim. Sci.* 84 2735–2742. 10.2527/jas.2005-414 16971575

[B219] Oakley B. B. Lillehoj H. S. Kogut M. H. Kim W. K. Maurer J. J. Pedroso A. (2014). The chicken gastrointestinal microbiome. *FEMS Microbiol. Lett.* 360 100–112. 10.1111/1574-6968.12608 25263745

[B220] O’Bryan C. A. Crandall P. G. Chalova V. I. Ricke S. C. (2008). Orange essential oils antimicrobial activities against *Salmonella* spp. *J. Food Sci.* 73 M264–M267. 19241555 10.1111/j.1750-3841.2008.00790.x

[B221] O’Bryan C. A. Pendleton S. J. Crandall P. G. Ricke S. C. (2015). Potential of plant essential oils and their components in animal agriculture–in vitro studies on antibacterial mode of action. *Front. Vet. Sci.* 2:35 10.3389/fvets.2015.00035 PMC4672195 26664964

[B222] Overhage J. Steinbuchel A. Priefert H. (2003). Highly efficient biotransformation of eugenol to ferulic acid and further conversion to vanillin in recombinant strains of *Escherichia coli*. *Appl. Environ. Microbiol.* 69 6569–6576. 14602615 10.1128/AEM.69.11.6569-6576.2003 PMC262297

[B223] Overhage J. Steinbüchel A. Priefert H. (2006). Harnessing eugenol as a substrate for production of aromatic compounds with recombinant strains of *Amycolatopsis* sp. HR167. *J. Biotechnol.* 125 369–376. 10.1016/j.jbiotec.2006.03.024 16677732

[B224] Oyen L. P. A. Dung N. X. (1999). “Essential-oil plants,” in *Plant Resources of South East Asia*, eds Oyen L. P. A. Dung N. X. (Leiden: Backhuys Publishers), 15–49.

[B225] Özek K. Wellmann K. T. Ertekin B. Tarım B. (2011). Effects of dietary herbal essential oil mixture and organic acid preparation on laying traits, gastrointestinal tract characteristics, blood parameters and immune response of laying hens in a hot summer season. *J. Anim. Feed Sci.* 20 575–586. 10.22358/jafs/66216/2011

[B226] Pan K. Chen H. Davidson P. M. Zhong Q. (2014). Thymol nanoencapsulated by sodium caseinate: physical and antilisterial properties. *J. Agric. Food Chem.* 62 1649–1657. 10.1021/jf4055402 24484459

[B227] Papatsiros V. G. Katsoulos P. D. Koutoulis K. C. Karatzia M. Dedousi A. Christodoulopoulos G. (2013). Alternatives to antibiotics for farm animals. *CAB Rev.* 8 1–15.

[B228] Parker C. T. Miller W. G. Horn S. T. Lastovica A. J. (2007). Common genomic features of *Campylobacter jejuni* subsp. *doylei* strains distinguish them from *C. jejuni* subsp. *jejuni*. *BMC Microbiol.* 7:50. 10.1186/1471-2180-7-50 17535437 PMC1892558

[B229] Parker C. T. Quiñones B. Miller W. G. Horn S. T. Mandrell R. E. (2006). Comparative genomic analysis of *Campylobacter jejuni* strains reveals diversity due to genomic elements similar to those present in *C. jejuni* strain RM1221. *J. Clin. Microbiol.* 44 4125–4135. 10.1128/jcm.01231-06 16943349 PMC1698300

[B230] Parkhill J. Wren B. W. Mungall K. Ketley J. M. Churcher C. Basham D. (2000). The genome sequence of the food-borne pathogen *Campylobacter jejuni* reveals hypervariable sequences. *Nature* 403 665–668. 10.1038/35001088 10688204

[B231] Parsons C. M. (1984). Influence of caecectomy and source of dietary fibre or starch on excretion of endogenous amino acids by laying hens. *Br. J. Nutr.* 51 541–548. 6326800 10.1079/bjn19840059

[B232] Perez-Vilar J. Hill R. L. (1999). The structure and assembly of secreted mucins. *J. Biol. Chem.* 274 31751–31754. 10.1074/jbc.274.45.31751 10542193

[B233] Phadtare S. Severinov K. (2010). RNA remodeling and gene regulation by cold shock proteins. *RNA Biol.* 7 788–795. 10.4161/rna.7.6.13482 21045540 PMC3073336

[B234] Piskernik S. Klanènik A. Riedel C. T. Brøndsted L. Možina S. S. (2011). Reduction of *Campylobacter jejuni* by natural antimicrobials in chicken meat-related conditions. *Food Control* 22 718–724. 10.1016/j.foodcont.2010.11.002

[B235] Piva G. Rossi F. (1998). “Possible alternatives to the use of antibiotics as growth promoters: new additives,” in *Proceedings of the IFIF II Conference of Mixed–Feed Manufactures in the Mediterranean*, Reus, 83–106.

[B236] Prior R. L. Cao G. (2000). Analysis of botanicals and dietary supplements for antioxidant capacity: a review. *J. AOAC Int.* 83 950–956. 10995120

[B237] Quaroni A. Isselbacher K. J. Ruoslahti E. (1978). Fibronectin synthesis by epithelial crypt cells of rat small intestine. *Proc. Natl. Acad. Sci. U.S.A.* 75 5548–5552. 10.1073/pnas.75.11.5548 103096 PMC393003

[B238] Raut J. S. Karuppayil S. M. (2014). A status review on the medicinal properties of essential oils. *Ind. Crops Prod.* 62 250–264. 10.1016/j.indcrop.2014.05.055

[B239] Ravindran V. Bryden W. L. (1999). Amino acid availability in poultry—In vitro and in vivo measurements. *Aust. J. Agric. Res.* 50 889–908.

[B240] Ricke S. C. (2003). The gastrointestinal tract ecology of *Salmonella* Enteritidis colonization in molting hens. *Poult. Sci.* 82 1003–1007. 10.1093/ps/82.6.1003 12817456

[B241] Ricke S. C. (2018). Impact of prebiotics on poultry production and food safety. *Yale J. Biol. Med.* 91 151–159. 29955220 PMC6020725

[B242] Ricke S. C. Dunkley C. S. Durant J. A. (2013). A review on development of novel strategies for controlling *Salmonella* Enteritidis colonization in laying hens: fiber-based molt diets. *Poult. Sci.* 92 502–525. 10.3382/ps.2012-02763 23300320

[B243] Ricke S. C. Van der Aar P. J. Fahey G. C. Jr. Berger L. L. (1982). Influence of dietary fibers on performance and fermentation characteristics of gut contents from growing chicks. *Poult. Sci.* 61 1335–1343. 10.3382/ps.0611335

[B244] Rimini S. Petracci M. Smith D. P. (2014). The use of thyme and orange essential oils blend to improve quality traits of marinated chicken meat. *Poult. Sci.* 93 2096–2102. 10.3382/ps.2013-03601 24902698

[B245] Robbe C. Capon C. Coddeville B. Michalski J. C. (2004). Structural diversity and specific distribution of O-glycans in normal human mucins along the intestinal tract. *Biochem. J.* 384 307–316. 10.1042/bj20040605 15361072 PMC1134114

[B246] Rosenquist H. Nielsen N. L. Sommer H. M. Norrung B. Christensen B. B. (2003). Quantitative risk assessment of human campylobacteriosis associated with thermophilic *Campylobacter* species in chickens. *Int. J. Food Microbiol.* 83 87–103. 10.1016/s0168-1605(02)00317-3 12672595

[B247] Rossi P. G. Bao L. Luciani A. Panighi J. Desjobert J. M. Costa J. (2007). (E)-Methylisoeugenol and elemicin: antibacterial components of *Daucus carota* L. essential oil against *Campylobacter jejuni*. *J. Agric. Food Chem.* 55 7332–7336. 10.1021/jf070674u 17685629

[B248] Roto S. M. Rubinelli P. M. Ricke S. C. (2015). An introduction to the avian gut microbiota and the effects of yeast-based prebiotic-type compounds as potential feed additives. *Front. Vet. Sci.* 2:28. 10.3389/fvets.2015.00028 26664957 PMC4672232

[B249] Ruberto G. Baratta M. T. Deans S. G. Dorman H. D. (2000). Antioxidant and antimicrobial activity of *Foeniculum vulgare* and *Crithmum maritimum* essential oils. *Planta Med.* 66 687–693. 10.1055/s-2000-9773 11199122

[B250] Rubinchik S. Seddon A. Karlyshev A. V. (2012). Molecular mechanisms and biological role of *Campylobacter jejuni* attachment to host cells. *Eur. J. Microbiol. Immunol.* 2 32–40. 10.1556/EuJMI.2.2012.1.6 24611119 PMC3933988

[B251] Ryu J. H. Beuchat L. R. (2005). Biofilm formation by *Escherichia coli* O157: H7 on stainless steel: effect of exopolysaccharide and curli production on its resistance to chlorine. *Appl. Environ. Microbiol.* 71 247–254. 10.1128/aem.71.1.247-254.2005 15640194 PMC544232

[B252] Sahin O. Kassem I. I. Shen Z. Lin J. Rajashekara G. Zhang Q. (2015). *Campylobacter* in poultry: ecology and potential interventions. *Avian Dis.* 59 185–200. 10.1637/11072-032315-Review 26473668

[B253] Sanders S. Q. Frank J. F. Arnold J. W. (2008). Temperature and nutrient effects on *Campylobacter jejuni* attachment on multispecies biofilms on stainless steel. *J. Food Prot.* 71 271–278. 10.4315/0362-028x-71.2.271 18326175

[B254] Santini C. Baffoni L. Gaggia F. Granata M. Gasbarri R. Di Gioia D. (2010). Characterization of probiotic strains: an application as feed additives in poultry against *Campylobacter jejuni*. *Int. J. Food Microbiol.* 141 S98–S108. 10.1016/j.ijfoodmicro.2010.03.039 20452074

[B255] Scallan E. Hoekstra R. M. Mahon B. E. Jones T. F. Griffin P. M. (2015). An assessment of the human health impact of seven leading foodborne pathogens in the United States using disability adjusted life years. *Epidemiol. Infect.* 143 2795–2804. 10.1017/S0950268814003185 25633631 PMC9151020

[B256] Scher K. Romling U. Yaron S. (2005). Effect of heat, acidification, and chlorination on *Salmonella enterica* serovar Typhimurium cells in a biofilm formed at the air-liquid interface. *Appl. Environ. Microbiol.* 71 1163–1168. 10.1128/aem.71.3.1163-1168.2005 15746314 PMC1065136

[B257] Schets F. M. Jacobs-Reitsma W. F. van der Plaats R. Q. Heer L. K. D. van Hoek A. H. Hamidjaja R. A. (2017). Prevalence and types of *Campylobacter* on poultry farms and in their direct environment. *J. Water Health* 15 849–862. 10.2166/wh.2017.119 29215350

[B258] Sergeant M. J. Constantinidou C. Cogan T. A. Bedford M. R. Penn C. W. Pallen M. J. (2014). Extensive microbial and functional diversity within the chicken cecal microbiome. *PLoS One* 9:e91941. 10.1371/journal.pone.0091941 24657972 PMC3962364

[B259] Shin E. Hong H. Oh Y. Lee Y. (2015). First report and molecular characterization of a *Campylobacter jejuni* isolate with extensive drug resistance from a travel-associated human case. *Antimicrob. Agents Chemother.* 59 6670–6672. 10.1128/aac.01395-15 26239988 PMC4576103

[B260] Si W. Gong J. Tsao R. Zhou T. Yu H. Poppe C. (2006). Antimicrobial activity of essential oils and structurally related synthetic food additives towards selected pathogenic and beneficial gut bacteria. *J. Appl. Microbiol.* 100 296–305. 10.1111/j.1365-2672.2005.02789.x 16430506

[B261] Sibanda N. McKenna A. Richmond A. Ricke S. C. Callaway T. Stratakos A. C. (2018). A review of the effect of management practices on *Campylobacter* prevalence in poultry farms. *Front. Microbiol.* 9:2002. 10.3389/fmicb.2018.02002 30197638 PMC6117471

[B262] Sikkema J. de Bont J. A. Poolman B. (1995). Mechanisms of membrane toxicity of hydrocarbons. *Microbiol. Rev.* 59 201–222. 7603409 10.1128/mr.59.2.201-222.1995 PMC239360

[B263] Silva J. Leite D. Fernandes M. Mena C. Gibbs P. A. Teixeira P. (2011). *Campylobacter* spp. as a foodborne pathogen: a review. *Front. Microbiol.* 2:200. 10.3389/fmicb.2011.00200 21991264 PMC3180643

[B264] Sima F. Stratakos A. C. Ward P. Linton M. Kelly C. Pinkerton L. (2018). A novel natural antimicrobial can reduce the *in vitro* and *in vivo* pathogenicity of T6SS positive *Campylobacter jejuni* and *Campylobacter coli* chicken isolates. *Front. Microbiol.* 9:2139. 10.3389/fmicb.2018.02139 30245680 PMC6137164

[B265] Skandamis P. Koutsoumanis K. Nychas G. J. E. Fasseas K. (2001). Inhibition of oregano essential oil and EDTA on *Escherichia coli* O157: H7 [food hygiene]. *Ital. J. Food Sci.* 13:65.

[B266] Smithard R. (2002). “Chapter 14: secondary plant metabolites in poultry nutrition,” in *Poultry Feedstuffs: Supply, Composition and Nutritive Value*, eds McNab J. M. Boorman K. N. (Wallingford, CT: CABI Publishing), 237–278. 10.1079/9780851994642.0237

[B267] Smith-Palmer A. Stewart J. Fyfe L. (1998). Antimicrobial properties of plant essential oils and essences against five important food-borne pathogens. *Lett. Appl. Microbiol.* 26 118–122. 10.1046/j.1472-765x.1998.00303.x 9569693

[B268] Son I. Englen M. D. Berrang M. E. Fedorka-Cray P. J. Harrison M. A. (2007). Prevalence of *Arcobacter* and *Campylobacter* on broiler carcasses during processing. *Int. J. Food Microbiol.* 113 16–22. 10.1016/j.ijfoodmicro.2006.06.033 16979251

[B269] Srey S. Jahid I. K. Ha S. D. (2013). Biofilm formation in food industries: a food safety concern. *Food Control* 31 572–585. 10.1016/j.foodcont.2012.12.001

[B270] Stahl M. Butcher J. Stintzi A. (2012). Nutrient acquisition and metabolism by *Campylobacter jejuni*. *Front. Cell. Infect. Microbiol.* 2:5 10.3389/fcimb.2012.00005 PMC3417520 22919597

[B271] Stahl M. Friis L. M. Nothaft H. Liu X. Li J. Szymanski C. M. (2011). L-fucose utilization provides *Campylobacter jejuni* with a competitive advantage. *Proc. Natl. Acad. Sci. U.S.A.* 108 7194–7199. 10.1073/pnas.1014125108 21482772 PMC3084102

[B272] Stanley D. Hughes R. J. Moore R. J. (2014). Microbiota of the chicken gastrointestinal tract: influence on health, productivity and disease. *Appl. Microbiol. Biotechnol.* 98 4301–4310. 10.1007/s00253-014-5646-2 24643736

[B273] Stern N. J. (2008). *Salmonella* species and *Campylobacter jejuni* cecal colonization model in broilers. *Poult. Sci.* 87 2399–2403. 10.3382/ps.2008-00140 18931193

[B274] Stern N. J. Fedorka-Cray P. Bailey J. S. Cox N. A. Craven S. E. Hiett K. L. (2001). Distribution of *Campylobacter* spp. in selected US poultry production and processing operations. *J. Food Prot.* 64 1705–1710. 10.4315/0362-028x-64.11.1705 11726147

[B275] Stern N. J. Svetoch E. A. Eruslanov B. V. Perelygin V. V. Mitsevich E. V. Mitsevich I. P. (2006). Isolation of a *Lactobacillus salivarius* strain and purification of its bacteriocin, which is inhibitory to *Campylobacter jejuni* in the chicken gastrointestinal system. *Antimicrob. Agents Chemother.* 50 3111–3116. 10.1128/aac.00259-06 16940109 PMC1563535

[B276] Szczepanski S. Lipski A. (2014). Essential oils show specific inhibiting effects on bacterial biofilm formation. *Food Control* 36 224–229. 10.1016/j.foodcont.2013.08.023

[B277] Thanissery R. Kathariou S. Smith D. P. (2014). Rosemary oil, clove oil, and a mix of thyme-orange essential oils inhibit *Salmonella* and *Campylobacter in vitro*. *J. Appl. Poult. Res.* 23 221–227. 10.3382/japr.2013-00888

[B278] Thanissery R. Smith D. P. (2014). Marinade with thyme and orange oils reduces *Salmonella* Enteritidis and *Campylobacter coli* on inoculated broiler breast fillets and whole wings. *Poult. Sci.* 93 1258–1262. 10.3382/ps.2013-03697 24795320

[B279] Thibodeau A. Fravalo P. Gauthier R. Guévremont E. Bergeron N. Laurent-Lewandowski S. (2014). Modification of *Campylobacter jejuni* broiler colonization by a feed additive composed of encapsulated organic acids and essential oils. *J. Agric. Sci. Technol. A* 4 853–864.

[B280] Thibodeau A. Fravalo P. Yergeau É. Arsenault J. Lahaye L. Letellier A. (2015). Chicken caecal microbiome modifications induced by *Campylobacter jejuni* colonization and by a non-antibiotic feed additive. *PLoS One* 10:e0131978. 10.1371/journal.pone.0131978 26161743 PMC4498643

[B281] Thomas M. T. Shepherd M. Poole R. K. van Vliet A. H. Kelly D. J. Pearson B. M. (2011). Two respiratory enzyme systems in *Campylobacter jejuni* NCTC 11168 contribute to growth on L-lactate. *Environ. Microbiol.* 13 48–61. 10.1111/j.1462-2920.2010.02307.x 20653766

[B282] Tiihonen K. Kettunen H. Bento M. H. L. Saarinen M. Lahtinen S. Ouwehand A. C. (2010). The effect of feeding essential oils on broiler performance and gut microbiota. *Br. Poult. Sci.* 51 381–392. 10.1080/00071668.2010.496446 20680873

[B283] Troxell B. Petri N. Daron C. Pereira R. Mendoza M. Hassan H. M. (2015). Poultry body temperature contributes to invasion control through reduced expression of *Salmonella* pathogenicity island 1 genes in *Salmonella enterica* serovars Typhimurium and Enteritidis. *Appl. Environ. Microbiol.* 81 8192–8201. 10.1128/AEM.02622-15 26386070 PMC4651079

[B284] Tschech A. Schink B. (1985). Fermentative degradation of resorcinol and resorcylic acids. *Arch. Microbiol.* 143 52–59. 10.1007/bf00414768

[B285] Tsuneki H. Ma E. L. Kobayashi S. Sekizaki N. Maekawa K. Sasaoka T. (2005). Antiangiogenic activity of β-eudesmol *in vitro* and *in vivo*. *Eur. J. Pharmocol.* 512 105–115. 10.1016/j.ejphar.2005.02.035 15840394

[B286] Tu Q. V. McGuckin M. A. Mendz G. L. (2008). *Campylobacter jejuni* response to human mucin MUC2: modulation of colonization and pathogenicity determinants. *J. Med. Microbiol.* 57 795–802. 10.1099/jmm.0.47752-0 18566135

[B287] Turnberg L. A. (1987). Gastric mucus, bicarbonate and pH gradients in mucosal protection. *Clin. Invest. Med.* 10 178–180. 3304748

[B288] Turner J. R. (2009). Intestinal mucosal barrier function in health and disease. *Nat. Rev. Immunol.* 9 799–809. 10.1038/nri2653 19855405

[B289] U.S Food and Drug Administration [FDA] (2018). *GRAS Substances (SCOGS) Database.*

[B290] Ultee A. Bennik M. H. J. Moezelaar R. (2002). The phenolic hydroxyl group of carvacrol is essential for action against the food-borne pathogen *Bacillus cereus*. *Appl. Environ. Microbiol.* 68 1561–1568. 10.1128/aem.68.4.1561-1568.2002 11916669 PMC123826

[B291] Umaraw P. Prajapati A. Verma A. K. Pathak V. Singh V. P. (2017). Control of *Campylobacter* in poultry industry from farm to poultry processing unit: a review. *Crit. Rev. Food Sci. Nutr.* 57 659–665. 10.1080/10408398.2014.935847 25898290

[B292] Upadhyay A. Arsi K. Wagle B. R. Upadhyaya I. Shrestha S. Donoghue A. M. (2017). Trans-cinnamaldehyde, carvacrol, and eugenol reduce *Campylobacter jejuni* colonization factors and expression of virulence genes *in vitro*. *Front. Microbiol.* 8:713. 10.3389/fmicb.2017.00713 28487683 PMC5403884

[B293] van der Aar P. J. Fahey G. C. Jr. Ricke S. C. Allen S. E. Berger L. L. (1983). Effects of dietary fibers on mineral status of chicks. *J. Nutr.* 113 653–661. 10.1093/jn/113.3.653 6298389

[B294] van der Wielen P. W. Biesterveld S. Notermans S. Hofstra H. Urlings B. A. van Knapen F. (2000). Role of volatile fatty acids in development of the cecal microflora in broiler chickens during growth. *Appl. Environ. Microbiol.* 66 2536–2540. 10.1128/aem.66.6.2536-2540.2000 10831435 PMC110578

[B295] Van Deun K. Haesebrouck F. Van Immerseel F. Ducatelle R. Pasmans F. (2008). Short-chain fatty acids and L-lactate as feed additives to control *Campylobacter jejuni* infections in broilers. *Avian Pathol.* 37 379–383. 10.1080/03079450802216603 18622853

[B296] Van Immerseel F. Fievez V. De Buck J. Pasmans F. Martel A. Haesebrouck F. (2004). Microencapsulated short-chain fatty acids in feed modify colonization and invasion early after infection with *Salmonella* Enteritidis in young chickens. *Poult. Sci.* 83 69–74. 10.1093/ps/83.1.69 14761086

[B297] Vandamme P. Dewhirst F. E. Paster B. J. On S. L. W. (2006). *Bergey’s Manual of Systematic Bacteriology: The Proteobacteria (Part C)*, 2nd Edn Vol. 2 eds Garrity G. Brenner D. J. Staley J. T. Krieg N. R. Boone D. R. DeVos P. (Berlin: Springer Science & Business Media), 1147–1160.

[B298] Vegge C. S. Brøndsted L. Li Y. P. Bang D. D. Ingmer H. (2009). Energy taxis drives *Campylobacter jejuni* toward the most favorable conditions for growth. *Appl. Environ. Mmicrobiol.* 75 5308–5314. 10.1128/AEM.00287-09 19542337 PMC2725471

[B299] Velayudhan J. Jones M. A. Barrow P. A. Kelly D. J. (2004). L-serine catabolism via an oxygen-labile L-serine dehydratase is essential for colonization of the avian gut by *Campylobacter jejuni*. *Infect. Immun.* 72 260–268. 10.1128/iai.72.1.260-268.2004 14688104 PMC343963

[B300] Velayudhan J. Kelly D. J. (2002). Analysis of gluconeogenic and anaplerotic enzymes in *Campylobacter jejuni*: an essential role for phosphoenolpyruvate carboxykinase. *Microbiology* 148 685–694. 10.1099/00221287-148-3-685 11882702

[B301] Vidanarachchi J. K. Mikkelsen L. L. Sims I. M. Iji P. A. Choct H. (2005). Phytobiotics: alternatives to antibiotic growth promoters in monogastric animal feeds. *Recent Adv. Anim. Nutr.* 15 131–144.

[B302] Villemur R. (1995). Coenzyme A ligases involved in anaerobic biodegradation of aromatic compounds. *Can. J. Microbiol.* 41 855–861. 10.1139/m95-118 8590400

[B303] Wallace R. B. (2003). “Campylobacter,” in *Foodborne Microorganisms of Public Health Significance*, 6th Edn, ed. Hocking A. D. (Sydney: Australian Institute of Food Science and Technology), 311–331.

[B304] Wang G. Clark C. G. Taylor T. M. Pucknell C. Barton C. Price L. (2002). Colony multiplex PCR assay for identification and differentiation of *Campylobacter jejuni, C. coli, C. lari, C. upsaliensis*, and *C. fetus* subsp. *fetus*. *J. Clin. Microbiol.* 40 4744–4747. 12454184 10.1128/JCM.40.12.4744-4747.2002 PMC154608

[B305] Wang X. Farnell Y. Z. Peebles E. D. Kiess A. S. Wamsley K. G. S. Zhai W. (2016). Effects of prebiotics, probiotics, and their combination on growth performance, small intestine morphology, and resident *Lactobacillus* of male broilers. *Poult. Sci.* 95 1332–1340. 10.3382/ps/pew030 26944975

[B306] Wang Y. H. Avula B. Nanayakkara N. D. Zhao J. Khan I. A. (2013). Cassia cinnamon as a source of coumarin in cinnamon-flavored food and food supplements in the United States. *J. Agric. Food Chem.* 61 4470–4476. 10.1021/jf4005862 23627682

[B307] Wei A. Shibamoto T. (2007). Antioxidant activities and volatile constituents of various essential oils. *J. Agric. Food Chem.* 55 1737–1742. 10.1021/jf062959x 17295511

[B308] Wei S. Morrison M. Yu Z. (2013). Bacterial census of poultry intestinal microbiome. *Poult. Sci.* 92 671–683. 10.3382/ps.2012-02822 23436518

[B309] Wenk C. (2003). Herbs and botanicals as feed additive in monogastric animals. *Asian Australas. J. Anim. Sci.* 16 282–289. 10.5713/ajas.2003.282

[B310] White P. L. Baker A. R. James W. O. (1997). Strategies to control *Salmonella* and *Campylobacter* in raw poultry products. *Sci. Tech.* 16 525–541. 10.20506/rst.16.2.1046 9501366

[B311] Wilkinson N. Hughes R. J. Aspden W. J. Chapman J. Moore R. J. Stanley D. (2016). The gastrointestinal tract microbiota of the Japanese quail, *Coturnix japonica*. *Appl. Microbiol. Biotechnol.* 100 4201–4209. 10.1007/s00253-015-7280-z 26758298

[B312] Williams P. Losa R. (2001). The use of essential oils and their compounds in poultry nutrition. *Worlds Poult. Sci. J.* 17 14–15.

[B313] Windisch W. Kroismayr A. (2006). *The Effects of Phytobiotics on Performance and Gut Function in Monogastrics.* Cape Town: World Nutrition Forum, 85–90.

[B314] Windisch W. Kroismayr A. (2007). Natural phytobiotics for health of young piglets and poultry: mechanisms and application. *Poult. Sci.* 86:643.

[B315] Woodward C. L. Kwon Y. M. Kubena L. F. Byrd J. A. Moore R. W. Nisbet D. J. (2005). Reduction of *Salmonella enterica* serovar Enteritidis colonization and invasion by an alfalfa diet during molt in Leghorn hens. *Poult. Sci.* 84 185–193. 10.1093/ps/84.2.185 15742953

[B316] World Health Organization [WHO] (2013). *The Global View of Campylobacteriosis: Report of an Expert Consultation.* Utrecht: World Health Organization.

[B317] Wright J. A. Grant A. J. Hurd D. Harrison M. Guccione E. J. Kelly D. J. (2009). Metabolite and transcriptome analysis of *Campylobacter jejuni* in vitro growth reveals a stationary-phase physiological switch. *Microbiology* 155 80–94. 10.1099/mic.0.021790-0 19118349

[B318] Xu J. Zhou F. Ji B. P. Pei R. S. Xu N. (2008). The antibacterial mechanism of carvacrol and thymol against *Escherichia coli*. *Lett. Appl. Microbiol.* 47 174–179. 10.1111/j.1472-765X.2008.02407.x 19552781

[B319] Yousaf M. S. Ahmad I. Ashraf K. Rashid M. A. Hafeez A. Ahmad A. (2017). Comparative effects of different dietary concentrations of β-galacto-oligosaccharides on serum biochemical metabolites, selected caecel microbiota and immune response in broilers. *J. Anim. Plant Sci.* 27 98–105.

[B320] Zemek J. Košíková B. Augustin J. Joniak D. (1979). Antibiotic properties of lignin components. *Folia Microbiol.* 24 483–486. 10.1007/bf02927180 389763

[B321] Ziprin R. L. Young C. R. Stanker L. H. Hume M. E. Konkel M. E. (1999). The absence of cecal colonization of chicks by a mutant of *Campylobacter jejuni* not expressing bacterial fibronectin-binding protein. *Avian Dis.* 43 586–589. 10494431

[B322] Zoetendal E. G. Collier C. T. Koike S. Mackie R. I. Gaskins H. R. (2004). Molecular ecological analysis of the gastrointestinal microbiota: a review. *J. Nutr.* 134 465–472. 10.1093/jn/134.2.465 14747690

[B323] Zviely M. (2004). “Aromatic chemicals,” in *Kirk-Othmer Encyclopedia of Chemical Technology*, 5th Edn, ed. Seidel A. (New York, NY: Wiley Interscience),226–262.

